# Common mental disorders in Peruvian immigrant in Chile: a comparison with the host population

**DOI:** 10.1186/s12889-023-15793-7

**Published:** 2023-06-30

**Authors:** Antonia Errazuriz, Kristin Schmidt, Paulina Valenzuela, Rodolfo Pino, Peter B. Jones

**Affiliations:** 1 Department of Psychiatry, School of Medicine, Pontificia Universidad Catolica de Chile, Diagonal Paraguay 362, Santiago, 8330077 Chile; 2 Department of Psychiatry and Mental Health, University of Concepción, Concepción, Chile; 3 Santiago, Chile; 4 Department of Psychiatry, University of Cambridge, Cambridge, UK; 5 NIHR Applied Research Collaboration East of England, Cambridge, UK

**Keywords:** Common mental disorders, Depression, Anxiety, Immigrant mental health, Healthy immigrant effect

## Abstract

**Background:**

The *Inner Santiago Health Study* (ISHS) aimed to (i) estimate the prevalence of common mental disorders (CMD; i.e. depressive and anxiety disorders) among immigrants of Peruvian origin in Chile; (ii) determine whether such immigrants are at higher risk of CMD when compared with the native-born geographically matched population (i.e. non-immigrants); and (iii) identify factors associated with higher risk of *any CMD* among this immigrant group. A secondary aim was to describe access to mental health services by Peruvian immigrants meeting criteria for *any CMD*.

**Methods:**

Findings are based on a population-based cross-sectional household mental health survey of 608 immigrant and 656 non-immigrant adults (18-64 years) residing in Santiago de Chile. Diagnoses of ICD-10 depressive and anxiety disorders and of *any CMD* were obtained using the Revised Clinical Interview Schedule. The relationships between demographic, economic, psychosocial, and migration-specific predictor variables, and risk of *any CMD* were analyzed with a series of stepwise multivariate logistic regression models.

**Results:**

The one-week prevalence of *any CMD* was 29.1% (95% CI: 25.2-33.1) among immigrants and 34.7% (95% CI: 30.7-38.7) among non-immigrants. Depending on the statistical model used in the pooled sample, we found the prevalence of *any CMD* among non-immigrants to be higher (OR=1.53; 95% CI: 1.05-2.25) or similar (OR=1.34; 95% CI: 0.94-19.2) when compared with immigrants. In the multivariate stepwise regression of *any CMD* in immigrants only, the prevalence was higher for females, those with primary compared to higher education, in debt and exposed to discrimination. Conversely, higher levels of functional social support, sense of comprehensibility, and manageability were associated with a lower risk of *any CMD* in immigrants. In addition, no differences were observed between immigrants and non-immigrants reporting *any CMD* in mental health service utilization.

**Conclusion:**

Our results evidence high levels of current CMD in this immigrant group, particularly amongst women. However, lower adjusted prevalence of *any CMD* in immigrants compared to non-immigrants was limited to preliminary statistical models, thus failing to provide clear support for a “healthy immigrant effect”. The study sheds new light on differences in CMD prevalence by immigrant status in Latin America by examining differential exposure to risk factors in immigrant versus non-immigrant groups.

**Supplementary Information:**

The online version contains supplementary material available at 10.1186/s12889-023-15793-7.

## Background 

Despite a large body of research, the effects of migration on mental health remain unclear. Some studies report a “healthy immigrant effect” [1], referring to findings of better physical and mental health in international immigrants compared with non-immigrants in the host country [2] and with those who stay behind in their country of origin [3]. For depressive and anxiety disorders (i.e. common mental disorders; CMD), lower prevalence has been observed among immigrants compared with non-immigrants in Northern American [4], and European [5–7] contexts, as well as in New Zealand [8]. Collectively, immigrants experiencing better mental health despite often being exposed to a number of risk factors has been labelled a health paradox [9]. Nevertheless, in some cases the migration experience and associated stressors appear to have a negative impact on mental health [10, 11]. For example, higher prevalence of CMD is reported among Mexican immigrants in the US versus non-immigrants in Mexico [12–14]. Moreover, and in contrast to CMD, much evidence suggests that the migrant experience is associated with increased risk of lower-prevalence, psychotic disorders [15].

A potential explanation for findings of better mental health in immigrants despite their exposure to risk factors lies in the health and psychological profiles of those choosing to migrate: individuals who choose to migrate have been shown to have better pre-migration mental health [16], healthier lifestyles and lower rates of chronic illness [17], and may therefore also be more competitive in selection processes inherent to migration [18, 19]. On the other hand, evidence suggests risk factors for poorer mental health in immigrants include barriers to successful integration in the host country, problems accessing health services [20], lower experience of social support and exposure to discrimination [21], and employment in higher-risk occupations [22]. Results further appear to differ by specific immigrant groups [6, 18] immigrants’ age at migration [23], and strata such as generational status [9, 12] (eTables 1 and 2) and sex [19, 24]. Finally, macro- and microeconomic factors play a role, with economic instability in the host country [25] and income poverty [26] are linked to higher prevalence of depression among immigrants, while better economic conditions in the host country (measured using Gross National Product) are systematically associated with lower prevalence [27]. Finally, lower prevalence of both depressive and anxiety disorders has also been observed for immigrants from high-income countries (HICs) compared with those from low-and middle-income countries (LMICs) [18, 19].

Given that research stems almost exclusively from HICs, and the associated lack of evidence from upper- and lower-middle-income countries (UMIC and LMIC, respectively [24]), it is difficult to assess the generalizability of the findings on the relation between risk vs. protective factors and CMD among immigrants. One region with extremely scarce data is Latin America, which has recently seen an unprecedented growth in predominantly intra-continental migration [28] from LMICs to host countries with better economic conditions. These countries often share an official language and many cultural elements, in stark contrast to many of the migration contexts described in the literature pertaining to HICs described above. The available regional research suggests that immigrants who migrate during childhood or adolescence have higher estimates of depressive mood and disorder compared with those migrating later in life [29–31]. Perceived discrimination has also been reported as a risk factor, with higher levels associated with more symptoms of anxiety and depression among Haitian immigrants in Brazil [32], and with more mental health problems in Venezuelan refugees and immigrants in Peru [33].

One of the Latin American countries increasingly receiving economic immigrants is Chile, which has been defined as a HIC since 2013 but was previously (and including at the time of this study’s fieldwork) classed as an UMIC. In 2017, Chile had an estimated number of 700,000 immigrants, representing 4.4% of the total population [34]. A large majority of these arrived within the previous 10 years, and over 85% of them originate from Latin America, with Peruvians forming the largest group (25.2% of all immigrants [34]). The little available data suggests prevalence estimates of CMD to be similar in the general populations of Chile [35, 36] and Peru [37]. However, Peru (classed as an UMIC) presents higher morbidity indicators, and lower levels of health resources, access, and coverage compared with Chile [38]. Given the dearth of data, the need for evidence on mental health status and service use among recent migrants to Chile is pressing. In a singular comparative study in primary care, Peruvian immigrants show lower prevalence of CMD compared with Chilean non-immigrants [39], despite more recent evidence identifying barriers in access to healthcare across immigrant groups [40]. Finally, and consistent with the international literature, perceived discrimination is associated with increased mental health problems and symptoms of depression/anxiety among economic immigrants of Peruvian and Colombian origin in Chile [41, 42].

To further address the lack of mental health data associated with Latin American migration, we report findings from a population-based cross-sectional household mental health survey conducted in adult residents of two inner-city districts of the Santiago Metropolitan Region (Región Metropolitana; RM). This study (the *Inner Santiago Health Study*; ISHS) aimed to: (i) estimate the prevalence of CMD (i.e. depression and anxiety disorders) among Peruvian immigrants (who form the largest immigrant group by nationality in Chile) (i.e. immigrants); (ii) determine whether immigrants are at higher risk of CMD when compared with the native-born geographically matched population (i.e. non-immigrants); and (iii) identify demographic, economic, psychosocial, and migration-specific factors associated with higher risk of CMD among immigrants. A secondary aim was to describe access to mental health services by Peruvian immigrants who met criteria for *any CMD *.

## Methods

### Design and sample

This study is a population-based cross-sectional household mental health survey of first-generation adult Peruvian immigrants (18-64 years) and geographically matched Chilean non-immigrants. It was approved by the University of Cambridge Psychology Research Ethics Committee (UK; No. 2010.70) and the Ethics Committee of the Metropolitan Northern Health Area of Santiago RM (Chile). At the time of data collection in 2011, the two selected inner city districts of Santiago RM, Recoleta and central Santiago, reported the first and third greatest number of Peruvian-born immigrants in Chile [43], representing 2.6% of the area’s adult population. They were chosen to control for between-groups socioeconomic differences. Full details of the study design, sampling procedures and data collection are reported elsewhere [44]. Following a pilot study and training of experienced interviewers, questionnaires were administered in face-to-face interviews.

Per district, the sampling framework of the Chilean National Institute of Statistics was used, with (1) primary sampling units (PSUs): clusters of 200 households on average; (2) secondary sampling units (SSUs): individual households within each cluster, and (3) final sampling units: eligible household members (eFigure 1). Multi-stage random probability sampling was used, comparable to that of other household survey designs conducted in developing countries [45]. To be eligible, participants had to (1) be a community-residing adult (18-64 years), (2) be able to read and write, and (3) have been born in Peru (immigrants) or Chile (non-immigrants). Participants were excluded if they reported (1) a disability or condition that made participation difficult; (2) political refugee status, or (3) having lived 12 or more months outside of Chile (non-immigrants only). The overall response rate was 61.1% (eFigure 2).

## Measures

### Psychiatric morbidity outcomes 

The Chilean version of the Revised Clinical Interview Schedule (CIS-R) [46], a lay-interviewer structured clinical interview, was used to asses psychiatric morbidity. The CIS-R has generally shown high specificity but moderate sensitivity [47], and has been extensively used in community studies with good reliability and validity in the Chilean population [36]. It monitors the presence of 14 symptoms during the past 7 days (psychosomatic symptoms, fatigue, concentration/forgetfulness, sleep problems, irritability, worry about physical health, depressive mood, depressive ideas, general worry, free-floating anxiety, phobias, panic, compulsions, and obsessions). Based on these, the application of an algorithm enables the diagnosis of seven CMD according to ICD-10 research diagnostic criteria [48]: depressive episode (f32.00, f32.01, f32.1, f32.11 or f32.2), panic disorder (f41.0), generalized anxiety disorder (GAD) (f41.1), obsessive–compulsive disorder (OCD) (f42.0), agoraphobia (f40.00, f40.01), social phobia (40.1), and specific phobia (f40.2). For this study, agoraphobia, social phobia, and specific phobia were grouped into *any phobia* and panic disorder, GAD, OCD, and any phobia were grouped into the diagnostic category *any anxiety disorder*. Non-specific psychiatric morbidity was identified using a cut-off of 12 or more points on the CIS-R for individuals that failed to meet any of the specific ICD-10 diagnoses above. This category approximately corresponds to the ICD-10 criterion of mixed anxiety and depressive disorder (MADD) (f41.2). Finally, cases of *any CMD* were defined as meeting criteria for ICD-10 any anxiety disorder, depressive episode, or MADD and the total CIS-R score was considered indicative of severity of symptoms. Participants were also asked if they had visited a mental health professional (psychiatrist, non-psychiatrist specialist, general practitioner, psychologist, and other health professionals) during the last 3 months for emotional or mental health reasons.

### Demographic, economic, and psychosocial factors

Participants’ sex, age, district of residence, number of children aged 18 or under, highest educational level (primary: 8 years or less; secondary: 9 to 12 years; or higher: > 12 years), employment status (unemployed or economically inactive versus employed), personal/breadwinner´s occupation (manual or non-manual), per capita income (in Chilean pesos), and current debt status (with or without) were recorded. Formal employment (with or without contract) was measured among employed participants not reporting self-employment.

Exposure to community violence was assessed based on a positive response to the participant or a household member having been a victim of burglary within the last 12 months using items from the Chilean National Security Survey [49]. Furthermore, participants completed three self-report questionnaires: 1) the 8-item Spanish version [50] of the Duke-UNC Functional Social Support Questionnaire (FSSQ) [51]; 2) four items relating to the trust and cohesion aspects from the Peruvian version of the Short Adapted Social Capital Assessment Tool (SASCAT) [52]; and 3) the 13-item Spanish version of the Orientation to Life Questionnaire (OLQ-13) [53], assessing three sense of coherence sub-factors: comprehensibility (the perception of events as making logical sense), manageability (the feeling of being able to cope), and meaningfulness (the feeling of life making sense and challenges being worthy of commitment). Higher scores on these measures indicate a stronger sense of each concept.

### Migration-specific variables 

Participants were classed as immigrants if born in Peru and non-immigrants if born in Chile. Immigrants were asked about their legal migration status (nationalized or legal resident, applying for residency, non-resident not processing visa) and length of stay in Chile (short: 0-4, medium: 5-9, or long: 10+ years). Age at immigration was calculated according to arrival during childhood (age 12 or under), adolescence (ages 13 to 19), or adulthood (age 20 or older), based on cut-offs from US studies [54]. Immigrants also reported their previous employment status in Peru (employed versus unemployed/inactive) immediately before migrating to Chile. Self-evaluated mismatch between expectations and achievements following migration (not at all/poorly satisfied or partially/perfectly achieved) in five domains (work, income, health family and friends) was assessed using items from the Bologna Migration History and Social Integration Interview [55].

Discrimination was assessed using three questions translated and adapted from the EMPIRIC study [56]: (i) “Have you ever been refused a job for reasons which you think were to do with your nationality?” (ii) “Have you ever been treated unfairly at work with regards to a promotion or move to a better position for reasons which you think were to do with your nationality?” (iii) “In the last twelve months, has anyone insulted you for reasons to do with your nationality?” An additive score of the positive responses to the three items was calculated.

### Statistical analysis

Analyses were performed in R (version 4.0.2). Based on non-normal distributions for all continuous variables in the immigrant and non-immigrant samples (as assessed with the Lilliefors (Kolmogorov-Smirnov) test), descriptive statistics were calculated as percentages, medians, and interquartile ranges (IQR). Between-group differences in categorical variables were calculated using chi-square tests, differences of proportions in each level using two-proportion z-tests with Yates correction, and differences in continuous variable using Wilcoxon rank sum tests.

Data were weighted to account for the age and sex distribution of the Peruvian and Chilean-born populations residing in the catchment area using the Chilean 2002 Census data (eTable 3). Overall and sex-stratified weighted prevalence estimates for psychiatric morbidity and weighted percentages of mental health service use (both with 95% confidence intervals) were calculated separately for immigrants and non-immigrants. Group differences between immigrants and non-immigrants on outcome variables of psychiatric morbidity and mental health service utilization were analyzed using multivariate logistic regression models with logit link, adjusting for age and sex.

In the immigrant group and in the pooled sample, independent associations between each predictor and risk of *any CMD* were analyzed with odds ratios and 95% confidence intervals based on univariate logistic regression analysis with logit link. The relationships between predictor variables and risk of *any CMD* were analyzed with a series of multivariate logistic regression models in the immigrant and pooled samples. Stepwise (backward and forward) methodology was used to select the multivariate regression model that best predicted the risk of *any CMD*. Model selection was based on two quality criteria: i) the lowest Akaike information criterion (AIC), and ii) the highest area under the ROC curve value (calculated by splitting the sample 80/20 into train and test subsamples). All evaluations of statistical significance are based on two-sided tests using the 5% level of significance.

## Results

### Sample characteristics

To identify private households with at least one person aged 18 to 64 born in Chile or Peru, a total of 2,573 SSUs were visited. The final sample of 1,264 adults successfully interviewed consisted of 608 Peruvian-born immigrants and 656 Chilean non-immigrants. Table 1 presents characteristics of the sample. Compared with non-immigrants, immigrants were younger, more likely to have three or more children, less likely to have attained primary or higher education and more likely to have completed secondary education. Participants classed as immigrants, particularly men, were more likely to be employed than non-immigrants (94% of immigrant *vs* 75.9% of non-immigrant men; 66.7% of immigrant *vs* 59.1% of non-immigrant women). Immigrants in the present study were also more likely than non-immigrants to be performing manual occupations at the time of the survey and while they reported lower levels of per capita income, they were also less likely to currently hold debt. Immigrants and non-immigrants did not differ in level of formal employment, exposure to community violence, perceived functional social support or cognitive social capital. On the sub-factors of their sense of coherence, immigrants and non-immigrants reported similar levels of comprehensibility and manageability, while immigrants reported higher levels for meaningfulness. Of the Peruvian-born participants, 79.9% had arrived in Chile as adults (aged 20+ years), 68.6% had resided in the country for at least 5 years, 96.5% were legal residents or processing/renewing their visa, and 76.9% reported being employed in Peru before migration. Most immigrants considered that their expectations about work (84.7%), income (85.7%), family (83.9%), health (88.0%) and friend (83.1%) were partially or perfectly achieved (eTable 5) and 50.2% reported at least one event of discrimination in the last year.

**Table 1:** Sample characteristics and differences by immigrant status

	Immigrants (n=608)	Non-immigrants (n=656)
	n (%) or Median (IQR)	n (%) or Median (IQR)
Demographic		
Sex		
Male	275 (45.2)	288 (43.9)
Female	333 (54.8)	368 (56.1)
Age (years)	34.0 (28.0 to 41.0)	41.0 (28.0 to 53.0)
District		
Recoleta	266 (43.8)	318 (48.5)
Santiago	342 (56.2)	338 (51.5)
Number of children		
< 3	547 (90.0)	614 (93.6)
3 or +	61 (10.0)	42 (6.4)
Economic		
Education level		
Primary	53 (8.7)	120 (18.3)
Secondary	398 (65.5)	329 (50.2)
Higher	157 (25.8)	207 (31.6)
Currently employed, yes	481 (79.6)	432 (66.1)
Formal employment, yes	279 (66.0)	208 (62.5)
Manual occupation, yes	445 (73.9)	260 (42.6)
Per capita income (CLP)	116,667 (75,000-173,333)	125,000 (75,625-206,667)
Active debt, yes	186 (30.6)	356 (54.4)
Psychosocial		
Experience of community violence, yes	206 (33.9)	234 (35.8)
Functional social support (Duke-UNC)^a^	34.0 (28.0 to 39.0)	35.0 (27.0 to 39.0)
Cognitive social capital (0-4) (SASCAT)^b^	3.0 (1.0 to 4.0)	3.0 (1.0 to 4.0)
Sense of coherence (OLQ-13)^c^		
Comprehensibility	24.5 (20.0 to 29.0)	24.0 (19.0 to 28.0)
Manageability	18.0 (16.0 to 22.0)	18.0 (15.0 to 22.0)
Meaningfulness	22.0 (18.0 to 25.0)	21.0 (18.0 to 24.0)
Migration characteristics^†^		
Legal status		
Non-resident and not applying	21 (3.5)	
Applying for residency	40 (6.6)	
Legal resident/Nationalized	544 (89.9)	
Age at migration		
Child (0-12 years)	25 (4.1)	
Adolescent (13-19 years)	97 (16.0)	
Adult (20+ years)	486 (79.9)	
Length of stay		
Short (0-4 years)	191 (31.4)	
Medium (5-9 years)	200 (32.9)	
Long (10+ years)	217 (35.7)	
Employed before migration, yes	464 (76.9)	
Experience of discrimination (last 12 months), yes	302 (50.2)	

^a^Duke-UNC Functional Social Support Questionnaire (Duke-UNC); ^b^ Short Adapted Social Capital Assessment Tool (SASCAT); ^c^ Orientation to Life Questionnaire (OLQ-13)

^a-c^Higher scores indicate higher experience of indicated constructs

^†^Only assessed in the immigrant group

### Prevalence of psychiatric symptoms, CMD and mental health service use

The CIS-R showed good internal consistency (α ≥ 0.8; eTable 4), similar to the 0.88 reported in the original Chilean reliability study [46]. The one-week prevalence estimates for psychiatric symptoms are presented in Table 2. In both groups, the most reported symptoms were general worry, fatigue, and irritability. Comparing immigrants versus non-immigrants, no significant differences were observed in the overall prevalence of psychosomatic symptoms, depressive mood, depressive ideas, general worry, phobias, panic, compulsions, or obsessions after adjusting for age and sex (all *p*>0.05). However, immigrants were less likely to report symptoms of fatigue, concentration/memory problems (*p*<0.001), sleep problems (*p*<0.001), irritability (*p*<0.001) and free-floating anxiety (*p*=0.010), and more likely to report worries about physical health (*p*=0.002) than Chilean-born participants.

**Table 2:** Estimated one-week percentage prevalence of CIS-R symptoms by immigrant status

Symptom	Immigrants (n=608)			Non-immigrants (n=656)				
	n	Prevalence %	(95% CI)	n	Prevalence %	(95% CI)	OR (95% CI) ^†^	p value
Psychosomatic	132	22.3	(18.9-25.7)	179	25.2	(21.8-28.6)	1.25 (0.95-1.65)	0.115
Fatigue	196	34.2	(30.2-38.1)	277	41.6	(37.6-45.6)	1.47 (1.15-1.89)	0.002
Concentration/forgetfulness	79	13.8	(11.0-16.7)	138	20.5	(17.3-23.7)	1.80 (1.31-2.48)	<0.001
Sleep problems	102	17.3	(14.2-20.4)	201	29.9	(26.2-33.5)	2.05 (1.54-2.73)	<0.001
Irritability	138	24.8	(21.2-28.4)	210	32.4	(28.6-36.1)	1.73 (1.32-2.26)	<0.001
Worry about physical health	40	6.8	(4.8-8.9)	17	2.7	(1.4-4.1)	0.39 (0.21-0.69)	0.002
Depressive mood	93	16.9	(13.7-20.1)	134	20.4	(17.1-23.6)	1.31 (0.96-1.79)	0.087
Depressive ideas	102	18.7	(15.4-22.0)	142	21.9	(18.5-25.2)	1.32 (0.98-1.79)	0.072
General worry	230	39.5	(35.5-43.5)	286	43.1	(39.1-47.0)	1.26 (0.99-1.60)	0.059
Free-floating anxiety	87	15.3	(12.3-18.3)	134	21	(17.7-24.3)	1.51 (1.10-2.07)	0.01
Phobias	39	7.3	(5.1-9.6)	52	7.6	(5.5-9.6)	1.30 (0.83-2.05)	0.257
Panic	35	6	(4.1-8.0)	36	4.9	(3.2-6.5)	0.90 (0.54-1.51)	0.697
Compulsions	74	12.7	(9.9-15.4)	103	15	(12.2-17.8)	1.35 (0.96-1.90)	0.084
Obsessions	48	8.1	(5.9-10.4)	77	11.3	(8.8-13.9)	1.48 (0.99-2.21)	0.055

Frequencies unweighted, percentages and 95% Confidence Intervals (CI) weighted for age and sex

^†^ Adjusted for age and sex (ref: immigrants)

Abbreviation: *CIS-R *Clinical Interview Schedule-Revised

One-week prevalence estimates for specific CMD are presented in Table 3. In both groups, the most common disorder was mixed anxiety and depressive disorder (MADD) with 20% (95% CI: 16.6-23.4) of non-immigrants and 16.8% (95% CI: 13.5-20.0) of immigrants meeting its criteria. This was followed by depressive episode with a prevalence of 6.2% (95% CI: 4.1-8.3) among immigrants and 7.0% (95% CI: 4.9-9.1) among non-immigrants. Specific anxiety disorders (phobias, generalized anxiety disorder (GAD), obsessive compulsive disorder (OCD), and panic disorder) had the lowest prevalence estimates; 2.4% (95% CI: 1.1-3.7), 2.1% (95% CI: 0.8-3.3), 3.7% (95% CI: 2.1-5.3) and 2.8% (95% CI: 1.4-4.1) in immigrants and 3.7% (95% CI: 2.2-5.1), 3.6% (95% CI: 2.1-5.6), 4.2% (95% CI: 2.6-5.8), and 1.2% (95% CI: 0.4-1.9) in non-immigrants respectively. The estimated weighted prevalence of *any CMD,* defined as meeting criteria for any of the specific ICD-10 diagnoses, was 29.1% (95% CI: 25.2-33.1) among immigrants and 34.7% (95% CI: 30.7-38.7) among non-immigrants (see Table 3). For any ICD-10 anxiety disorder, 10.0% (95% CI: 7.6-12.4) of non-immigrants and 8.6% (95% CI: 6.2-11.0) of immigrants met criteria. After adjusting for age and sex, immigrants were less likely than non-immigrants to meet criteria for *any CMD* (OR=1.47; 95% CI: 1.12-1.93; *p*=0.006) and immigrants were more likely to meet criteria for panic disorder (OR=0.39; 95% CI: 0.15-0.90; *p*=0.033). However, no between-group differences were observed in the prevalence of depressive episode, phobias, GAD, OCD, any anxiety disorder, or MADD (all *p*>0.05).

**Table 3:** Estimated one-week prevalence of CIS-R Common Mental Disorders (ICD-10) by sex and immigrant status

	Immigrants (n=608)			Non-immigrants (n=656)				
	n	Prevalence %	(95% CI)	n	Prevalence %	(95% CI)	OR(95% CI)^†^	*p *value
Women								
Depressive episode^a^	29	9.5	(6.2-12.8)	36	10.2	(6.9-13.6)	1.15 (0.67-1.99)	0.614
Any phobia^b^	11	3.3	(1.4-5.2)	20	5.5	(3.0-7.9)	1.77 (0.82-3.98)	0.153
Generalized anxiety disorder	9	2.8	(1.0-4.6)	17	4.9	(2.5-7.3)	1.81 (0.79-4.43)	0.172
Obsessive compulsive disorder	16	4.7	(2.5-7.0)	22	5.4	(3.0-7.7)	1.23 (0.61-2.53)	0.558
Panic disorder	13	3.6	(1.6-5.6)	7	1.8	(0.4-3.2)	0.38 (0.13-0.99)	0.054
Any anxiety disorder^c^	37	11.4	(7.9-14.9)	50	14.0	(10.2-17.8)	1.23 (0.76-1.98)	0.403
Mixed anxiety and depressive disorder^d^	64	21.7	(16.9-26.5)	77	23.2	(18.5-28.0)	1.06 (0.72-1.58)	0.769
Any CMD^e^	117	38.6	(33.1-44.2)	150	44.8	(39.2-50.4)	1.28 (0.92-1.78)	0.144
Men								
Depressive episode	2	0.8	(0.0-1.9)	9	3.6	(1.2-6.0)	4.78 (1.15-32.46)	0.053
Any phobia	3	1.0	(0.0-2.2)	6	1.8	(0.4-3.3)	1.52 (0.36-7.74)	0.575
Generalized anxiety disorder	2	0.9	(0.0-2.2)	6	2.4	(0.5-4.3)	3.31 (0.72-23.21)	0.154
Obsessive compulsive disorder	5	2.0	(0.2-3.8)	8	3.1	(0.9-5.2)	1.66 (0.52-5.70)	0.397
Panic disorder	4	1.5	(0.0-3.0)	2	0.5	(0.0-1.3)	0.44 (0.05-2.57)	0.379
Any anxiety disorder	11	4.3	(1.8-6.9)	16	5.9	(3.0-8.7)	1.44 (0.63-3.36)	0.391
Mixed anxiety and depressive disorder	22	9.0	(5.4-12.7)	41	16.6	(11.8-21.4)	1.92 (1.08-3.47)	0.028
Any CMD	34	14.0	(9.6-18.5)	61	24.2	(18.8-29.7)	1.98 (1.22-3.24)	0.006
All								
Depressive episode	31	6.2	(4.1-8.3)	45	7.0	(4.9-9.1)	1.39 (0.85-2.32)	0.196
Any phobia	14	2.4	(1.1-3.7)	26	3.7	(2.2-5.1)	1.72 (0.87-3.50)	0.125
Generalized anxiety disorder	11	2.1	(0.8-3.3)	23	3.6	(2.1-5.2)	2.09 (1.00-4.60)	0.055
Obsessive compulsive disorder	21	3.7	(2.1-5.3)	30	4.2	(2.6-5.8)	1.36 (0.75-2.51)	0.316
Panic disorder	17	2.8	(1.4-4.1)	9	1.2	(0.4-1.9)	0.39 (0.15-0.90)	0.033
Any anxiety disorder	48	8.6	(6.2-11.0)	66	10.0	(7.6-12.4)	1.28 (0.85-1.94)	0.246
Mixed anxiety and depressive disorder	86	16.8	(13.5-20.0)	118	20.0	(16.6-23.4)	1.28 (0.93-1.77)	0.133
Any CMD	151	29.1	(25.2-33.1)	211	34.7	(30.7-38.7)	1.47 (1.12-1.93)	0.006

^a^ Includes any depressive episode in ICD-10 (f32.00, f32.01, f32.1, f32.11 or f32.2)

^b^ Includes social (f40.1), specific (f40.2) and agoraphobia (f40.0)

^c^ Includes ICD-10 generalized anxiety disorder (f41.1), obsessive–compulsive disorder (f42.0), panic disorders (f41.0), any phobia (f40.00, f40.01, f40.1, f40.2)

^d^ Corresponds to ICD-10 f41.2 and is defined as a total CIS-R score greater or equal to 12 but not meeting criteria for any specific anxiety disorder (c) or depressive episode (a)

^e^ Includes ICD-10 depressive episode (as in a), any ICD-10 anxiety disorder (as in c) and ICD-10 mixed anxiety and depressive disorder (as in d)

Frequencies unweighted, percentages and 95% CI weighted for age and sex using 2002 Chilean Population Census

† Adjusted for age and sex (ref: immigrants)

Abbreviations: *CMD* Common Mental Disorder, *CIS-R *Clinical Interview Schedule-Revised

The prevalence estimates of *any CMD* in immigrants and non-immigrants described separately for women and men can be found in Table 3. Across both sexes, the most common disorder was *MADD*. No differences were observed between immigrant and non-immigrant women across any of the studied CMD after adjusting for age (all *p*>0.05). Among men, immigrants were less likely to meet criteria for *any CMD* (*p*=0.006) and *MDD* (*p*=0.028) than non-immigrants after adjusting for age. No differences were observed between immigrant and non-immigrant men in the adjusted prevalence of depressive episode, phobias, GAD, OCD, or panic disorder (all *p*>0.05). Within-group sex differences across diagnostic categories are reported in eTable 6.

Overall, immigrants were less likely than non-immigrant to have accessed mental health services in the last three months (*n*=37, 6.4% versus *n*=84, 11.2%, *p*<0.01). However, reported access was similar for immigrants (18.6%, 95% CI: 12.3-25.0) and non-immigrants (21.6%, 95% CI: 15.8-27.4) in those meeting criteria for *any CMD* (eTable 7). Finally, a positive association between severity of CIS-R symptoms and access was only observed among immigrants, but not among non-immigrants (eTable 8).

### Factors associated with any CMD

The internal consistency levels were excellent for the FSSQ (α ≥ 0.9), acceptable for the OLQ-13 (α ≥ 0.7), and questionable for the SASCAT items (α > 0.6) (eTable 4).

Logistic models of the univariate and multivariate associations between all demographic, economic and psychosocial factors, and the risk of *any CMD* in the pooled sample showed that immigrants had lower odds of *any CMD* compared with non-immigrants (see Table 4 for details). However, the association between immigrant status and *any CMD* was not significant in a stepwise multivariate model, which included fewer demographic, economic and psychosocial factors (OR=1.34; 95% CI: 0.94-1.92; *p*=0.101; Table 4). This stepwise model showed similar model evaluation criteria as the full multivariate model (full multivariate model: AIC = 862.9, AUC = 0.818; selective multivariate model: AIC = 945.7, AUC = 0.829).

**Table 4:** Factors associated with any Common Mental Disorder among residents of Santiago and Recoleta (*n*=1,264)

	Univariate logistic regression			Multivariate logistic regression					
				All variables^†^			Selected variables ^‡^		
	OR^¥^	(95% CI)	*p *value	OR	(95% CI)	*p* value	OR	(95% CI)	*p* value
Immigrant status (immigrant^§^ *vs* non-Immigrant)	1.48	(1.15-1.90)	0.002	1.53	(1.05-2.25)	0.028	1.34	(0.94-1.92)	0.101
Sex (male^§^ *vs* female)	3.15	(2.40-4.15)	<0.001	2.93	(2.01-4.30)	<0.001	3.07	(2.18-4.37)	<0.001
Age (18-27^§^)									
28-37	1.09	(0.77-1.56)	0.628	0.95	(0.58-1.57)	0.855	0.88	(0.56-1.40)	0.601
38-47	1.31	(0.90-1.92)	0.160	1.10	(0.63-1.92)	0.728	1.01	(0.61-1.67)	0.962
48+	1.51	(1.06-2.16)	0.023	1.06	(0.63-1.78)	0.825	1.04	(0.64-1.67)	0.887
District (Recoleta^§^ *vs* Santiago)	1.30	(1.01-1.67)	0.041	1.55	(1.09-2.23)	0.028	1.57	(1.13-2.19)	0.008
Number of children (<3^§^ *vs* 3+)	2.04	(1.31-3.15)	0.001	1.29	(0.69-2.41)	0.426			
Education level (higher 12+^§^)									
Primary (=<8 yrs)	2.70	(1.80-4.06)	<0.001	2.13	(1.18-3.86)	0.012	2.11	(1.21-3.68)	0.008
Secondary (9-12 yrs)	1.55	(1.15-2.10)	0.004	1.54	(1.02-2.32)	0.040	1.52	(1.04-2.23)	0.032
Employment status (employed^§^ *vs* unemployed/ economically inactive)	1.24	(0.94-1.64)	0.122	0.91	(0.60-1.37)	0.649			
Per capita income	1.00	(1.00-1.00)	0.001	1.00	(1.00-1.00)	0. 709			
Active debt (no^§^ *vs* yes)	2.14	(1.66-2.75)	<0.001	1.92	(1.34-2.75)	<0.001	1.93	(1.37-2.71)	<0.001
Experience of community violence (no^§^ *vs* yes)	2.09	(1.62-2.71)	<0.001	2.18	(1.53-3.13)	<0.001	2.09	(1.50-2.92)	<0.001
Functional social support^ƒ^	0.93	(0.91-0.94)	<0.001	0.96	(0.93-0.98)	0.001	0.96	(0.94-0.98)	<0.001
Cognitive social capital^ƒ^	0.79	(0.72-0.87)	<0.001	0.98	(0.86-1.11)	0.709			
Sense of coherence									
Comprehensibility^ƒ^	0.86	(0.84-0.88)	<0.001	0.91	(0.88-0.94)	<0.001	0.90	(0.87-0.93)	<0.001
Manageability^ƒ^	0.81	(0.78-0.84)	<0.001	0.90	(0.85-0.94)	<0.001	0.89	(0.85-0.94)	<0.001
Meaningfulness^ƒ^	0.86	(0.83-0.89)	<0.001	0.97	(0.93-1.02)	0.237			

§ Reference category

^¥^ Unadjusted

^ƒ^ Higher scores indicate higher experience of indicated constructs

^†^ n=942; AIC=862.9, C-statistic = 0.849; H&L = Chi-sq(8) 5.70 (p=0681); AUC = 0.818

^‡^ n=1,039; AIC=945.7; C-statistic = 0.842; H&L = Chi-sq(8) 6.02 (p=0.645); AUC = 0.829

Table 5 displays associations of demographic, economic, psychosocial and migration factors with the risk of *any CMD* in immigrants only. Univariate logistic regression analyses showed that among immigrants, prevalence of *any CMD* was significantly higher in females (*p*<0.001), residents of Santiago (*p*=0.028), participants with 3 or more children (*p*=0.030), those with only primary education (*p*=0.005 vs. higher education), unemployed or economically inactive (*p*=0.004), in debt (*p*<0.001), exposed to community violence (*p*<0.001). Those reporting lower levels of income (*p*<0.011), functional social support (*p*<0.001), cognitive social capital (*p*=0.024), and sense of coherence (subscales of comprehensibility, manageability, and meaningfulness, all *p*<0.001), as well as those unemployed or economically inactive in Peru (*p*<0.002) and exposure to discrimination (*p*<0.001) also showed higher risk of *any CMD*, while no association was found with age*.*

**Table 5:** Factors associated with any Common Mental Disorder among immigrants (*n*=608)

	Univariate logistic regression			Multivariate logistic regression					
				All variables^†^			Selected variables^‡^		
	OR^¥^	95% CI	*p* value	OR	95% CI	*p *value	OR	95% CI	*p *value
Sex (male^§^ *vs* female)	3.98	(2.62-6.19)	<0.001	2.98	(1.61-5.65)	0.001	3.69	(2.18-6.38)	<0.001
Age (18-27^§^)									
28-37	1.03	(0.63-1.69)	0.911	1.45	(0.60-3.58)	0.419	0.98	(0.51-1.87)	0.945
38-47	1.10	(0.64-1.89)	0.721	2.34	(0.79-7.11)	0.127	1.16	(0.57-2.37)	0.673
48+	1.02	(0.53-1.92)	0.962	1.53	(0.44-5.34)	0.502	0.98	(0.41-2.29)	0.958
District (Recoleta^§^ *vs* Santiago)	1.54	(1.05-2.27)	0.028	1.64	(0.94-2.90)	0.084	1.58	(0.96-2.62)	0.074
Number of children (< 3^§^ *vs* 3+)	1.92	(1.05-3.44)	0.030	1.30	(0.50-3.27)	0.586			
Education level (higher 12+^§^)									
Primary (=< 8 yrs)	2.63	(1.33-5.20)	0.005	2.30	(0.84-6.30)	0.103	2.48	(1.01-6.14)	0.047
Secondary (9-12 yrs)	1.30	(0.83-2.07)	0.262	1.78	(0.94-3.44)	0.080	1.51	(0.85-2.72)	0.165
Employment status (employed^§^ *vs *unemployed/economically inactive)	1.90	(1.22-2.94)	0.004	1.69	(0.81-3.54)	0.167			
Per capita income	1.00	(1.00-1.00)	0.011	1.00	(1.00-1.00)	0.998			
Active debt (no^§^ *vs* yes)	2.19	(1.48-3.25)	<0.001	1.96	(1.07-3.58)	0.028	2.02	(1.22-3.38)	0.007
Experience of community violence (no^§^ *vs* yes)	1.97	(1.34-2.91)	<0.001	1.79	(1.00-3.24)	0.051			
Functional social support^ƒ^	0.92	(0.90-0.94)	<0.001	0.94	(0.91-0.97)	<0.001	0.95	(0.92-0.98)	0.003
Cognitive social capital^ƒ^	0.84	(0.72-0.98)	0.024	1.12	(0.90-1.40)	0.303			
Sense of coherence									
Comprehensibility^ƒ^	0.87	(0.84-0.90)	<0.001	0.90	(0.84-0.95)	<0.001	0.89	(0.85-0.94)	<0.001
Manageability^ƒ^	0.82	(0.78-0.86)	<0.001	0.90	(0.83-0.98)	0.015	0.92	(0.86-0.98)	0.018
Meaningfulness^ƒ^	0.87	(0.83-0.90)	<0.001	0.98	(0.92-1.05)	0.628			
Age at migration (as child/adolescent (0-12^§^)									
Adolescent (13-19 years)	0.97	(0.37-2.79)	0.959	1.00	(0.24-4.45)	0.998			
Adult (20+ years)	0.84	(0.35-2.23)	0.704	0.67	(0.14-3.42)	0.626			
Length of stay (short^§^)									
Medium	1.18	(0.73-1.92)	0.504	1.14	(0.55-2.40)	0.722			
Long	1.47	(0.93-2.36)	0.105	1.13	(0.48-2.66)	0.784			
Employment status in Peru (employed^§^ *vs* unemployed/economically inactive)	1.98	(1.29-3.01)	0.002	1.65	(0.81-3.36)	0.168			
Events of discrimination (0^§^ *vs* 1+)	2.37	(1.61-3.51)	<0.001	2.67	(1.49-4.89)	0.001	2.34	(1.42-3.93)	0.001

§ Reference category

¥ Unadjusted

ƒ Higher scores indicate higher experience of indicated constructs

† n=446; AIC=404.3; C-statistic = 0.86; H&L = Chi-sq(8) 5.30 (p=0.725); AUC = 0.802

‡ n=496; AIC=437.7; C-statistic = 0.843; H&L = Chi-sq(8) 6.56 (p=0.181); AUC = 0.810

The best-fitting multivariate stepwise model of *any CMD* in immigrants showed that the prevalence was significantly higher for females (OR=3.69; 95% CI: 2.18-6.38; *p*<0.001), those with primary compared with higher education (OR=2.48; 95% CI: 1.01-6.14; *p*=0.047), those with active debt (OR=2.02; 95% CI: 1.22-3.38; *p*=0.007), and those exposed to discrimination (OR=2.34; 95% CI: 1.42-3.93; *p*=0.001). Lower levels of functional social support (OR=0.95; 95% CI: 0.92-0.98; *p*=0.003), sense of comprehensibility (OR=0.89; 95% CI: 0.85-0.94; *p*<0.001), and manageability (OR=0.92; 95% CI: 0.86-0.98; *p*=0.018) were also associated with a higher risk of *any CMD* in immigrants (Table 5).

## Discussion

To our knowledge, this is the first population-based study to investigate the prevalence of CMD among Peruvian immigrants residing in private households of Santiago RM, Chile. Overall, we found CMD, as measured with the CIS-R, to be highly prevalent among immigrants, particularly in women. In a multivariate model with all predictive variables included, the prevalence of *any CMD* was significantly lower in Peruvians than in geographically matched Chileans, but this healthy immigrant effect was not supported by a selective stepwise regression. Given the models’ similar predictive power, our findings on the association between immigrant status and CMD therefore remain inconclusive. On average, immigrants were younger, had higher education levels, more children, were less frequently in debt, and reported higher levels of meaningfulness than non-immigrants. They also reported low levels of mismatch between expectations and achievements across the domains of work, income, health, family, and friends. Although a larger proportion of immigrants were employed at the time of data collection, they were also more likely to work in manual occupations and reported lower per capita income. Similar levels of psychosocial vulnerability across immigrants and non-immigrants were found, based on exposure to informal employment, community violence, perceived social support, cognitive social capital, and sense of coherence. Key factors associated with higher risk of *any CMD* in immigrants were female sex, having only attained primary education, active debt, experience of community violence, perceived discrimination, and low functional social support, comprehensibility, and manageability.

The results of our full multivariate model show lower prevalence of *any CMD* in immigrants, which is in line with literature on the healthy immigrant effect [2]. However, this evidence is weak given that the effect was specific to *any CMD*, without group differences in prevalence estimates for *depressive episode*, *an*y *anxiety disorder,* or other diagnoses, and was not consistently found across both statistical models. Direct comparison with Chilean and regional prevalence of CMD is complicated by a scarcity of data, but one methodologically similar study in a sample of the general population of Santiago RM reported prevalence estimates of *depressive episode*, *any anxiety disorder,* and *any CMD* similar to those in our immigrant sample [36].

We found that only one in five Peruvian immigrants meeting criteria for *any CMD* accessed mental health care in the prior three months, suggesting a treatment gap between needs and professional support similar to the general population of other Latin American countries [57]. However, this gap did not differ in comparison with Chilean non-immigrants and was lower than previously reported in Chile [58]. The overall similar level of access to care among immigrant and non-immigrant participants with *any CMD* in this study may be interpreted in the light of the studied immigrant group’s characteristics, specifically, the high proportion of immigrants holding formal employment (ensuring access to the public National Healthcare System), the low level of perceived mismatch of expectations and achievements in the health domain (reflecting high satisfaction; eTable 5), and the previously mentioned large community of Peruvian immigrants, who face relatively low cultural and language barriers in the host country. However, immigrants with higher symptom severity did not access treatment more frequently, which indicates the need to develop a better understanding of the pathway to care and existing barriers among immigrants with CMD.

Our data suggest that it is difficult to generalize CMD outcomes based on immigrant status alone, and that stronger predictors may be found across psychosocial and psychological domains. While immigrants have reported increased exposure to risk factors such as poverty [26], high-risk occupations [22], and discrimination [59], the current study sample show a relatively low-risk migrant profile, including recent arrival, migration during adulthood, low levels of perceived mismatch between expectations and achievements, and – more generally – the large size of the Peruvian community in Chile and cultural assimilation may have acted as potential protective factors. Exposure to stressors previously associated with negative mental health in immigrants, such as illegal status [59] and cultural/linguistic barriers [17] was minimal. Furthermore, our sample showed high job market participation, which may have also facilitated access to the health system, a line of reasoning that is supported by comparable treatment gaps in the immigrant and non-immigrant samples [60]. Levels of social vulnerability levels were also similar across the immigrant and non-immigrant samples, as assessed by exposure to community violence, social support, cognitive social capital, and comprehensibility and manageability.

The one-week ICD-10 prevalence estimates of CMD among our immigrant sample were generally higher than those previously reported in the general population of Peru when comparing against one-month DSM-IV prevalence (using the Composite International Diagnostic Interview [CIDI]; World Mental Health Survey, WMHS [61]) and ICD-10 point prevalence (using the MINI International Neuropsychiatric Interview [MINI]; Peruvian Epidemiological Mental Health Studies [62–74]; eTable 9). When comparing our 1-week prevalence of depressive/any anxiety disorders with Latin American studies outside of Peru (eTable 10), we found estimates, specifically those for the female immigrant sample, to be higher than those in the general population of Mexico [75] but similar to 1-month ICD-10 prevalence in the general population of Brazil [76, 77], a country reporting comparatively high prevalence in the region [37]. In comparison with the general population of Colombia, female immigrants in our study reported a similar prevalence of depressive episode, while men reported a lower prevalence [78].

Across international epidemiological studies in the general population of both HICs [79–83] and LMICs [84, 85] using the same case definition and the CIS-R, the prevalence of CMD appears to be lower than observed in the present study (eTable 11). Our prevalence of *any CMD* also appears to be much higher than reported in a recent meta-analysis [86] of 12-month CMD across general populations which used a narrower definition of CMD not including MADD, defined in our study as cases scoring 12 or more but not meeting criteria for a specific ICD-10 diagnoses. Finally, based on meta-analytic findings that control for methodological factors or perform subgroup analysis for studies using interview-based assessment method, the point prevalence of depressive and anxiety disorders across general populations globally appears similar to our estimates for immigrants in the present study [87, 88]. Notably among immigrants, prevalence estimates for CMD for males were much lower than for females, an effect observable in many of the previous studies reported here but which can additionally be interpreted in the light of the extremely high labor force participation of Peruvian immigrant men in Chile.

A number of demographic, economic, and psychosocial factors predicted an increased risk of *any CMD* in the multivariate model specific to immigrants: namely, female sex, primary education, active debt, exposure to community violence, and self-reports of low social support and of low positive life orientation such as comprehensibility and manageability. Beyond the higher prevalence of CMD among immigrant women in the present study, which is consistent with finding across the international literature [86], the strongest predictors of increased risk of any CMD were low educational attainment and debt, indicators associated with economic disadvantage. The linear association between lower educational level and higher risk of any CMD replicates findings from the Santiago Mental Disorders Survey [36] and is consistent with findings from other Latin American studies [89]). There is also a well-established relationship between unsecured debt and physical and mental health [90], which is supported by our findings of active debt being related to higher prevalence of *any CMD*. Both primary education and debt had higher predictive value than employment and income, which should be explored further as specific dimensions of socioeconomic status that may increase risk of CMD in immigrants.

We also found a strong association between lower social support and higher risk of *any CMD* in immigrants, a finding consistent with a large body of evidence from general [90, 91] and immigrant [59] studies. Exposure to community violence has been reported as a risk factor for CMD in Colombia [92] and Brazil [93], and is replicated as such in the current results. Finally, our finding of sense of coherence as a protective factor for *any CMD* supports its stress-buffering effect in both immigrant [94] and general populations [95–97].

When examining migration-specific factors, the experience of discrimination within the last year was the strongest predictor of increased risk for *any CMD* in immigrants. Repeatedly found to be a risk-factor for negative health outcomes [98], CMD [99], and extending to other Latin American [33, 41, 42] and high income [59] settings, our results expand on the role of perceived discrimination as a risk factor for poorer health among economic immigrants in an upper middle-income setting. Contrary to our expectations, the risk of *any CMD* was not predicted by age at migration. This goes against extensive literature from the US and Canada demonstrating that migration during childhood or adolescence compared with adulthood poses a risk for poorer mental health outcomes [12]. Our lack of findings may be related to the demographic characteristics of the current sample, a minority of which had immigrated as children. This contrasts with migration experiences where exposure and the progressive acculturation to a greatly different host culture may be accompanied by a deterioration in mental health with increased length of stay (which may further confound age of migration effects).

Existing research on international migration shows vast methodological, case definition, and comparison-group differences across studies, which pose obstacles to drawing reliable parallels. Many assessments of depressive and anxiety symptoms/disorders are self-reports rather than based on diagnostic interviews, and the characterizations of immigrants (for example, as refugees or economic immigrants) and definitions of groups (such as immigrants and ethnic minorities) are not always clearly delineated. The same holds for comparison groups, as studies variably use second-generation immigrants of the same ethnicity, representative samples of the native population, or the general population of the country of origin. The development of further large-scale, methodologically rigorous epidemiological studies that allow regional and global comparisons of immigrants’ mental health should thus be of high priority.

This urban population-based cohort study of first-generation Peruvian immigrants and Chilean-born non-immigrants provides novel prevalence estimates from an understudied geographical region, where the acculturation process for the immigrant sample was shaped by high levels of cultural contiguity at the linguistic, religious, and ethnic level. We included a wide range of sociodemographic, economic, psychosocial, and migration-specific variables as potential predictors of higher risk of adverse mental health outcomes as measured with the CIS-R. We aimed to address common methodological issues with the use of well-validated instruments and a standardized clinical diagnostic tool (assessing depressive disorder, OCD, GAD, phobia, agoraphobia, panic disorder, and *any CMD*) that has previously been validated in the local population. Nevertheless, shortcomings of the study include the relatively low response rate (61.1%) compared with those reported in previous Chilean studies (90% in the Santiago Mental Disorders Survey [36]; 87.4% in Chile Psychiatric Prevalence Study [100]), the lack of data on selectiveness of participation by immigrants status, as well as its purely cross-sectional nature. The study's immigrant sample provides important evidence of the Latin American context, where immigration often takes place across neighboring and culturally similar countries; however, the data collection was carried out in 2011, and Chile has since seen drastic increases in the number of immigrants of both Peruvian and other nationalities (immigrants comprised 1.8% of the total population in 2010 and 4.4% in 2017 [34, 101]. Therefore, the generalization of our results to immigrant groups in divergent conditions remains limited. Specifically, this highlights the need for further research into sociodemographic differences such as prevalence estimates in immigrants with lower vs. higher educational level. More generally, academic studies would benefit from using longitudinal approaches to further understand the prevalence and severity of CMD over time (and potential causal mechanisms) for different immigrant subgroups. In the context of unprecedented levels of migration, local governments are also called upon to expand the monitoring of immigrant mental health by including relevant measures in health and social surveys.

## Conclusion 

Our study has showed a mental health advantage for Peruvian immigrants compared with Chilean non-immigrants only in estimates of non-specific CMD and preliminary regression models, thus providing limited evidence for a healthy immigrant effect. Amongst the pressing need for increased mental health service provision for CMD among both immigrants and non-immigrants, our findings specifically support a prioritization of resources towards potential protective factors, such as psychosocial interventions that strengthen individuals’ sense of coherence. Critically, and supported by strong associations between debt and discrimination, and higher risk of CMD in our study, any healthy immigrant effect may also be diluted, abolished, or reversed by the intersection with socioeconomic and psychosocial factors. This complexity must be considered in efforts by academic, financial and health organizations, and governments to support specific immigrant populations’ mental health.

## Supplementary Information

**Additional file 1.** 

## Data Availability

The de-identified dataset generated and analyzed during the current study is publicly available at Dataverse: Errazuriz, Antonia, 2022, "Inner Santiago Health Study (ISHS)", https://doi.org/10.7910/DVN/RLT7JI, Harvard Dataverse, V1.

## Abbreviations

CIS-R: Clinical Interview Schedule-Revised
CMD: Common Mental Disorders
GAD: General Anxiety Disorder
HIC: High income countries
ISHS: Inner Santiago Health Study
LMIC: Low- and middle-income countries
MADD: Mixed Anxiety and Depressive Disorder
OCD: Obsessive Compulsive Disorder
RM: Region Metropolitana
UMIC: Upper Middle-Income Country

## References

[1] Ichou M, Wallace M (2019). The healthy immigrant effect: the role of educational selectivity in the good health of migrants. Demograph Res.

[2] Aldridge RW, Nellums LB, Bartlett S, Barr AL, Patel P, Burns R, Hargreaves S, Miranda JJ, Tollman S, Friedland JS (2018). Global patterns of mortality in international migrants: a systematic review and meta-analysis. Lancet.

[3] Kennedy S, Kidd MP, McDonald JT, Biddle N (2015). The healthy immigrant effect: patterns and evidence from four countries. J Int Migration Integrat.

[4] Salas-Wright CP, Vaughn MG, Goings TC, Miller DP, Schwartz SJ (2018). Immigrants and mental disorders in the united states: new evidence on the healthy migrant hypothesis. Psychiatry Res.

[5] Markkula N, Lehti V, Gissler M, Suvisaari J (2017). Incidence and prevalence of mental disorders among immigrants and native Finns: a register-based study. Soc Psychiatry Psychiatr Epidemiol.

[6] de Wit MA, Tuinebreijer WC, Dekker J, Beekman AJ, Gorissen WH, Schrier AC, Penninx BW, Komproe IH, Verhoeff AP (2008). Depressive and anxiety disorders in different ethnic groups: a population based study among native Dutch, and Turkish, Moroccan and Surinamese migrants in Amsterdam. Soc Psychiatry Psychiatr Epidemiol.

[7] Dhadda A, Greene G (2018). 'The healthy migrant effect' for mental health in England: propensity-score matched analysis using the EMPIRIC survey. J Immigr Minor Health.

[8] Kokaua J, Schaaf D, Wells J, Foliaki S (2009). Twelve-month prevalence, severity, and treatment contact of mental disorders in New Zealand born and migrant Pacific participants in Te Rau Hinengaro: the New Zealand Mental Health Survey. Pacific Health Dialog.

[9] Marks A, Ejesi K, García Coll C: Understanding the U.S. Immigrant paradox in childhood and adolescence. Child Dev Perspect. 2014; 8:59-64.

[10] Bhugra D (2004). Migration and mental health. Acta Psychiatr Scand.

[11] Close C, Kouvonen A, Bosqui T, Patel K, O'Reilly D, Donnelly M (2016). The mental health and wellbeing of first generation migrants: a systematic-narrative review of reviews. Global Health.

[12] Breslau J, Borges G, Tancredi D, Saito N, Kravitz R, Hinton L, Vega W, Medina-Mora ME, Aguilar-Gaxiola S (2011). Migration from Mexico to the United States and subsequent risk for depressive and anxiety disorders: a cross-national study. Arch Gen Psychiatry.

[13] Lacey KK, Sears KP, Govia IO, Forsythe-Brown I, Matusko N, Jackson JS (2015). Substance use, mental disorders and physical health of Caribbeans at-home compared to those residing in the United States. Int J Environment Res Public Health.

[14] Orozco R, Borges G, Medina-Mora ME, Aguilar-Gaxiola S, Breslau J (2013). A cross-national study on prevalence of mental disorders, service use, and adequacy of treatment among Mexican and Mexican American populations. Am J Public Health.

[15] Jongsma HE, Turner C, Kirkbride JB, Jones PB (2019). International incidence of psychotic disorders, 2002–17: a systematic review and meta-analysis. Lancet Public Health.

[16] Donato KM, Caron L, Hamilton E: Migration and mental health in Mexico: domestic migrants, return U.S. migrants, and non-migrants. Front Psychiatry. 2020; 10:970-970. 10.3389/fpsyt.2019.00970 PMC7008711 32116812

[17] Lassetter J, Callister L (2009). The impact of migration on the health of voluntary migrants in western societies. J Transcult Nurs.

[18] Nakash O, Levav I, Gal G (2013). Common mental disorders in immigrant and second-generation respondents: results from the Israel-based world mental health survey. Int J Soc Psychiatry.

[19] Dalgard OS, Thapa SB, Hauff E, McCubbin M, Syed HR (2006). Immigration, lack of control and psychological distress: findings from the Oslo health study. Scand J Psychol.

[20] Sarría-Santamera A, Hijas-Gómez AI, Carmona R, Gimeno-Feliú LA (2016). A systematic review of the use of health services by immigrants and native populations. Pub Health Rev.

[21] Alegría M, Álvarez K, DiMarzio K (2017). Immigration and mental health. Current Epidemiology Reports.

[22] Schenker M (2010). A global perspective of migration and occupational health. Am J Ind Med.

[23] Breslau J, Borges G, Hagar Y, Tancredi D, Gilman S (2009). Immigration to the USA and risk for mood and anxiety disorders: variation by origin and age at immigration. Psychol Med.

[24] Meyer SR, Lasater M, Tol WA (2017). Migration and mental health in low- and middle-income countries: a systematic review. Psychiatry.

[25] Carta MG, Reda MA, Consul ME, Brasesco V, Cetkovich-Bakmans M, Hardoy MC (2006). Depressive episodes in Sardinian emigrants to Argentina: why are females at risk?. Soc Psychiatry Psychiatr Epidemiol.

[26] Bárcena-Martín E, Pérez-Moreno S (2017). Immigrant–native gap in poverty: a cross-national European perspective. Rev Econ Household.

[27] Lindert J, Ehrenstein OS, Priebe S, Mielck A, Brähler E (2009). Depression and anxiety in labor migrants and refugees–a systematic review and meta-analysis. Soc Sci Med.

[28] Pierola M, Rodriguez M: Migrants in Latin America: disparities in health status and in access to Healthcare. In. Edited by IDB-DP-00784 Dpn. Retrieved from: https://publications.iadb.org/publications/english/document/Migrants-in-Latin-America-Disparities-in-Health-Status-and-in-Access-to-Healthcare.pdf: Inter-American Development Bank, Washington DC; 2020. Accessed 23 May 2023.

[29] Andrade L, Wang Y, Andreoni S, Silveira C, Alexandrino-Silva C, Siu E, Nishimura R, Anthony JC, Gattaz WF, Kessler RC (2012). Mental disorders in megacities: findings from the são paulo megacity mental health survey, Brazil. PLoS One.

[30] Ruiz-Grosso P, Bernabe-Ortiz A, Diez-Canseco F, Gilman RH, Checkley W, Bennett IM, Miranda JJ, Group CCS (2015). Depressive mood among within-country migrants in Periurban Shantytowns of Lima, Peru. J Immigr Minor Health.

[31] Hernández-Vásquez A, Rojas-Roque C, Vargas-Fernández R, Bendezu-Quispe G (2020). Dynamics of depressive symptoms and within-country migration among Peruvian women. Rural Remote Health.

[32] Brunnet AE, Bolaséll LT, Weber JLA, Kristensen CH (2017). Prevalence and factors associated with PTSD, anxiety and depression symptoms in Haitian migrants in southern Brazil. Int J Soc Psychiatry.

[33] Mougenot B, Amaya E, Mezones-Holguin E, Rodriguez-Morales AJ, Cabieses B (2021). Immigration, perceived discrimination and mental health: evidence from Venezuelan population living in Peru. Globalization and Health.

[34] Instituto Nacional de Estadísticas: Características sociodemográficas de la inmigración internacional en Chile Censo 2017. 2018. In. Retrieved from http://www.censo2017.cl/descargas/inmigracion/181126-sintesis.pdf; Accessed 23 May 2023.

[35] Vicente B, Kohn R, Rioseco P, Saldivia S, Levav I, Torres S (2006). Lifetime and 12-month prevalence of DSM-III-R disorders in the Chile psychiatric prevalence study. Am J Psychiatry.

[36] Araya R, Rojas G, Fritsch R, Acuña J, Lewis G (2001). Common mental disorders in Santiago, Chile: prevalence and socio-demographic correlates. Br J Psychiatry.

[37] World Health Organization (2017). Depression and other common mental disorders: global health estimates.

[38] Pan American Health Organization: Health Situation in the Americas: Basic Health Indicators 2011. 2011. Retrieved from https://iris.paho.org/handle/10665.2/49353. Accessed 23 May 2023.

[39] Rojas G, Fritsch R, Castro A, Guajardo V, Torres P, Díaz B (2011). Mental disorders among immigrants in Chile. Rev Med Chil.

[40] Blukacz A, Cabieses B, Markkula N (2020). Inequities in mental health and mental healthcare between international immigrants and locals in Chile: a narrative review. Int J Equity Health.

[41] Lahoz S, Forns M (2016). Discriminación percibida, afrontamiento y salud mental en migrantes peruanos en Santiago de Chile. Psicoperspectivas Individuo y Sociedad.

[42] Urzúa A, Ferrer R, Godoy N, Leppes F, Trujillo C, Osorio C, Caqueo-Urízar A (2018). The mediating effect of self-esteem on the relationship between perceived discrimination and psychological well-being in immigrants. PLoS One.

[43] Instituto Nacional de Estadísticas: Censo de Población y Vivienda 2002. In. Santiago, Chile. Retrieved from https://www.ine.cl/estadisticas/sociales/censos-de-poblacion-y-vivienda/poblacion-y-vivienda. Accessed 23 May 2023.

[44] Errazuriz A: The Mental Health of Peruvian Immigrants in Santiago, Chile (doctoral thesis). Cambridge: Cambridge University; 2014. retrieved from 10.17863/CAM.63881.

[45] Pettersson H: Design of master sampling frames and master samples for household surveys in developing countries. In: Household surveys in developing and transition countries. edn. Edited by (Eds.) UDoEaSA. New York: United Nations. Retrieved from 10.17863/CAM.63881. 2005.

[46] Lewis G, Pelosi AJ, Araya R, Dunn G (1992). Measuring psychiatric disorder in the community: a standardized assessment for use by lay interviewers. Psychol Med.

[47] Taub NA, Morgan Z, Brugha TS, Lambert PC, Bebbington PE, Jenkins R, Kessler RC, Zaslavsky AM, Hotz T (2005). Recalibration methods to enhance information on prevalence rates from large mental health surveys. Int J Methods Psychiatr Res.

[48] World Health Organization: The ICD-10 Classification of Mental and Behavioural Disorders: Clinical descriptions and diagnostic guidelines. 1993.

[49] Instituto Nacional de Estadísticas: V Encuesta Nacional Urbana de Seguridad Ciudadana 2008: Retrieved from: http://www.seguridadciudadana.gob.cl/sitio-2010-2014/filesapp/ENUSC/ENUSC%202008/Cuestionario%20V%20Encuesta%20Nacional%20de%20de%20Victimizacion%202008.pdf. Accessed 23 May 2023.

[50] Bellón J, Delgado A, Luna J, Lardelli P (1996). Validez y fiabilidad del cuestionario de apoyo social funcional Duke-UNC-11. Atención Primaria.

[51] Broadhead WE, Gehlbach SH, de Gruy FV, Kaplan BH: The Duke-UNC Functional Social Support Questionnaire. Measurement of social support in family medicine patients. Med Care 1988, 26(7):709-723. 10.1097/00005650-198807000-00006 3393031

[52] De Silva MJ, Huttly SR, Harpham T, Kenward MG (2007). Social capital and mental health: a comparative analysis of four low income countries. Soc Sci Med.

[53] Moreno-Jiménez B, Alonso M, Álvarez E (1997). Sentido de coherencia, personalidad resistente, autoestima y salud. Revista de Psicología de la Salud.

[54] Breslau J, Aguilar-Gaxiola S, Borges G, Kendler KS, Su M, Kessler RC (2007). Risk for psychiatric disorder among immigrants and their US-born descendants: evidence from the National Comorbidity Survey Replication. J Nerv Ment Dis.

[55] Tarricone I, Atti AR, Braca M, Pompei G, Morri M, Poggi F, Melega S, Stivanello E, Tonti L, Nolet M (2011). Migrants referring to the Bologna transcultural psychiatric team: reasons for drop-out. Int J Soc Psychiatry.

[56] Weich S, McManus S: Common Mental Disorders: Norwich: The Stationery Office. Retrieved from: http://data.parliament.uk/DepositedPapers/Files/DEP2008-3141/DEP2008-3141.pdf. Accessed 23 May 2023.

[57] Kohn R, Ali AA, Puac-Polanco V, Figueroa C, López-Soto V, Morgan K, Saldivia S, Vicente B: Mental health in the Americas: an overview of the treatment gap. Rev Panam Salud Publica. 2018; 42:e165. 10.26633/RPSP.2018.165 PMC6386160 31093193

[58] Saldivia S, Vicente B, Kohn R, Rioseco P, Torres S (2004). Use of mental health services in Chile. Psychiatr Serv.

[59] Jurado D, Alarcón RD, Martínez-Ortega JM, Mendieta-Marichal Y, Gutiérrez-Rojas L, Gurpegui M (2017). Factors associated with psychological distress or common mental disorders in migrant populations across the world. Rev Psiquiatr Salud Ment.

[60] Organisation for Economic Co-operation and Development: Labour market outcomes of immigrants In: Indicators of Immigrant Integration 2015: Settling In. edn. Edited by OECD Publishing PEU, Brussels; 2015.

[61] Bromet E, Andrade L, Bruffaerts R, Williams D: Major Depressive Disorder. In: Mental Disorders Around the World: Facts and Figures from the WHO World Mental Health Surveys. edn. Edited by Scott KM, de Jonge P, Stein DJ, Kessler RC. Cambridge: Cambridge University Press; 2018. pp. 41-56.

[62] Instituto Especializado de Salud Mental: Estudio Epidemiológico en Salud Mental en la Selva Peruana 2004. Anales de Salud Mental 2005, XXI (Números 1 y 2), Peru. Retrieved from https://www.insm.gob.pe/investigacion/archivos/estudios/2004-ASM-EESM-SP/files/res/downloads/book.pdf. Accessed 23 May 2023.

[63] Instituto Especializado de Salud Mental: Estudio Epidemiológico de Salud Mental en Lima Rural 2007. Anales de Salud Mental 2008, XXIV (Números 1 y 2), Peru. Retrieved from http://www.insm.gob.pe/investigacion/archivos/estudios/2007-ASM-EESM-LR/files/res/downloads/book.pdf. Accessed 23 May 2023.

[64] Instituto Especializado de Salud Mental: Estudio Epidemiológico de Salud Mental en La Sierra Rural 2008. Anales de Salud Mental 2009, XXV (Números 1 y 2), Peru. Retrieved from https://cdn.www.gob.pe/uploads/document/file/389689/Estudio_epidemiol%C3%B3gico_de_salud_mental_en_la_sierra_rural_2008._Informe_general20191016-26158-b7ip7w.pdf. Accessed 23 May 2023.

[65] Instituto Especializado de Salud Mental: Estudio Epidemiológico de Salud Mental en la ciudad de Abancay 2010. Anales de Salud Mental 2011, XXVII (Suplemento 1), Peru. Retrieved from http://www.insm.gob.pe/investigacion/archivos/estudios/2010-asm-eesm-a/files/res/downloads/book.pdf. Accessed 23 May 2023.

[66] Instituto Especializado de Salud Mental: Estudio Epidemiológico de Salud Mental en la Selva Rural 2009. Anales de Salud Mental 2012, XXXVIII (Suplemento 2) Retrieved from http://www.insm.gob.pe/investigacion/archivos/estudios/2009-ASM-EESM-SR.pdf. Accessed 23 May 2023.

[67] Instituto Especializado de Salud Mental: Estudio Epidemiológico de Salud Mental en Lima Metropolitana y Callao-Replicación 2012. Anales de Salud Mental 2013, XXIX (Suplemento 1), Peru. Retrieved from http://www.insm.gob.pe/investigacion/archivos/estudios/2012%20ASM%20-EESM%20-LM.pdf. Accessed 23 May 2023.

[68] Instituto Especializado de Salud Mental: Estudio Epidemiológico de Salud Mental en la Ciudad de Huánuco 2013. Anales de Salud Mental 2016, XXXII (Número 2), Peru. Retrieved from https://www.insm.gob.pe/investigacion/archivos/estudios/2021/Vol%20XXXII%202016%20nro2%20EESM_ciudad%20de%20Huanuco%202013%20(1).pdf. Accessed 23 May 2023.

[69] Instituto Especializado de Salud Mental: Estudio Epidemiológico de Salud Mental en la Ciudad de Cerro de Pasco 2013. Anales de Salud Mental 2016, XXXII (Número 1), Peru. Retrieved from https://www.insm.gob.pe/investigacion/archivos/estudios/2021/Vol%20XXXII%202016%20nro1%20EESM_ciudad%20de%20Cerro%20de%20Pasco%202013%20(1).pdf. Accessed 23 May 2023.

[70] Instituto Especializado de Salud Mental: Estudio Epidemiológico de Salud Mental Comparativo Ciudad de Abancay 2016-2016. Informe General del Adulto. Anales de Salud Mental 2019, XXXV (Número 2), Peru. Retrieved from https://www.insm.gob.pe/investigacion/archivos/estudios/_notes/Vol%20XXXV%202019%20Nro2%20EESM_comparativo%20ciudad%20de%20Abancay%20%202010-2016.pdf. Accessed 23 May 2023.

[71] Instituto Especializado de Salud Mental: Estudio Epidemiológico Metropolitano de Salud 2002. Anales de Salud Mental 2002, XVIII (Números 1 y 2), Peru. Retrieved from http://www.insm.gob.pe/investigacion/archivos/estudios/2002-ASM-EESM-M/files/res/downloads/book.pdf. Accessed 23 May 2023.

[72] Instituto Especializado de Salud Mental: Estudio Epidemiológico de Salud Mental en La Sierra Peruana 2003. Anales de Salud Mental 2003, XIX (Números 1 y 2), Peru. Retrieved from https://www.insm.gob.pe/investigacion/archivos/estudios/2003-ASM-EESM-SP/files/res/downloads/book.pdf. Accessed 23 May 2023.

[73] Instituto Especializado de Salud Mental: Estudio Epidemiológico de Salud Mental en Fronteras 2005. Anales de Salud Mental 2006, XXII (Números 1 y 2), Peru. Retrieved from https://www.insm.gob.pe/investigacion/archivos/estudios/2005-ASM-EESM-F/files/res/downloads/book.pdf. Accessed 23 May 2023.

[74] Instituto Especializado de Salud Mental: Estudio Epidemiológico de Salud Mental en la Costa Peruana 2006. Anales de Salud Mental 2007, XXIII (Números 1 y 2), Peru. Retrieved from https://www.insm.gob.pe/investigacion/archivos/estudios/2006-ASM-EESM-CP/files/res/downloads/book.pdf. Accessed 23 May 2023.

[75] Medina-Mora ME, Borges G, Lara C, Benjet C, Jaimes J, Fleiz C, Villatoro Velazquez J, Guiot E, Ruíz J, Rodas L (2003). Prevalence of mental disorders and use of services: results from the Mexican national survey of psychiatric epidemiology. Salud Mental.

[76] Andrade L, Walters EE, Gentil V, Laurenti R (2002). Prevalence of ICD-10 mental disorders in a catchment area in the city of São Paulo. Brazil. Soc Psychiatry Psychiatr Epidemiol.

[77] Vorcaro CM, Lima-Costa MF, Barreto SM, Uchoa E (2001). Unexpected high prevalence of 1-month depression in a small Brazilian community: the Bambuí study. Acta Psychiatr Scand.

[78] Gómez-Restrepo C, Bohórquez A, Pinto Masis D, Gil Laverde JF, Rondón Sepúlveda M, Díaz-Granados N (2004). The prevalence of and factors associated with depression in Colombia. Rev Panam Salud Publica.

[79] McManus S, Bebbington P, Jenkins R, Brugha T: (eds.) Mental Health and Wellbeing in England: Adult Psychiatric Morbidity Survey 2014: The Health & Social Care Information Centre, Social Care Statistics; 2016. Retrieved from: https://www.gov.uk/government/statistics/adult-psychiatric-morbidity-survey-mental-health-and-wellbeing-england-2014. Accessed 23 May 2023.

[80] Lam LC, Wong CS, Wang MJ, Chan WC, Chen EY, Ng RM, Hung SF, Cheung EF, Sham PC, Chiu HF (2015). Prevalence, psychosocial correlates and service utilization of depressive and anxiety disorders in Hong Kong: the Hong Kong Mental Morbidity Survey (HKMMS). Soc Psychiatry Psychiatr Epidemiol.

[81] Skapinakis P, Bellos S, Koupidis S, Grammatikopoulos I, Theodorakis PN, Mavreas V (2013). Prevalence and sociodemographic associations of common mental disorders in a nationally representative sample of the general population of Greece. BMC Psychiatry.

[82] Singleton N, Bumpstead R, O'Brien M, Lee A, Meltzer H. Psychiatric morbidity among adults living in private households, 2000, vol. 15, 2003/05/15 edn. Retrieved from: https://www.researchgate.net/publication/266299241_Adult_psychiatric_morbidity_in_England_2007_Results_of_a_household_survey. Accessed 23 May 2023. 10.1080/0954026021000045967 12745312

[83] McManus S, Meltzer H, Brugha T, Bebbington P, Jenkins R: (eds.) Adult psychiatric morbidity in England, 2007: Results of a household survey.: The Health & Social Care Information Centre, Social Care Statistics; 2009. Retrieved from: https://files.digital.nhs.uk/publicationimport/pub02xxx/pub02931/adul-psyc-morb-res-hou-sur-eng-2007-rep.pdf. Accessed 23 May 2023.

[84] Jenkins R, Njenga F, Okonji M, Kigamwa P, Baraza M, Ayuyo J, Singleton N, McManus S, Kiima D (2012). Prevalence of common mental disorders in a rural district of Kenya, and socio-demographic risk factors. Int J Environment Res Pub Health.

[85] Krishnaswamy S, Subramaniam K, Jemain AA, Low WY, Ramachandran P, Indran T, Patel V (2012). Common mental disorders in Malaysia: Malaysian mental health survey, 2003–2005. Asia-Pacific Psychiatry.

[86] Steel Z, Marnane C, Iranpour C, Chey T, Jackson JW, Patel V, Silove D (2014). The global prevalence of common mental disorders: a systematic review and meta-analysis 1980–2013. Int J Epidemiol.

[87] Lim GY, Tam WW, Lu Y, Ho CS, Zhang MW, Ho RC (2018). Prevalence of depression in the community from 30 countries between 1994 and 2014. Sci Rep.

[88] Baxter AJ, Scott KM, Vos T, Whiteford HA (2013). Global prevalence of anxiety disorders: a systematic review and meta-regression. Psychol Med.

[89] Araya R, Lewis G, Rojas M, Fritsch R (2003). Education and income: which is more important for mental health?. J Epidemiol Commun Health.

[90] Richardson T, Elliott P, Roberts R (2013). The relationship between personal unsecured debt and mental and physical health: a systematic review and meta-analysis. Clin Psychol Rev.

[91] Santini ZI, Koyanagi A, Tyrovolas S, Mason C, Haro JM (2015). The association between social relationships and depression: a systematic review. J Affect Disord.

[92] Orrego S, Sierra-Hincapié G, Bernal D (2020). Mental disorders in the context of trauma and violence in a population study. Revista Colombiana de Psiquiatría (English ed).

[93] Cruz MS, Silva ES, Jakaite Z, Krenzinger M, Valiati L, Gonçalves D, Ribeiro E, Heritage P, Priebe S (2021). Experience of neighbourhood violence and mental distress in Brazilian favelas: a cross-sectional household survey. Lancet Reg Health Am.

[94] Erim Y, Morawa E, Atay H, Aygün S, Gökalp P, Senf W (2011). Sense of coherence and depression in the framework of immigration: Turkish patients in Germany and in Turkey. Int Rev Psychiatry.

[95] Gustavsson-Lilius M, Julkunen J, Keskivaara P, Lipsanen J, Hietanen P (2012). Predictors of distress in cancer patients and their partners: the role of optimism in the sense of coherence construct. Psychol Health.

[96] Konttinen H, Haukkala A, Uutela A (2008). Comparing sense of coherence, depressive symptoms and anxiety, and their relationships with health in a population-based study. Soc Sci Med.

[97] Carlén K, Suominen S, Lindmark U, Saarinen M, Aromaa M, Rautava P, Sillanpää M (2020). Sense of coherence predicts adolescent mental health. J Affective Disord.

[98] Pascoe EA, Smart Richman L (2009). Perceived discrimination and health: a meta-analytic review. Psychol Bull.

[99] Alegría M, NeMoyer A, Falgàs Bagué I, Wang Y, Alvarez K (2018). Social determinants of mental health: where we are and where we need to go. Curr Psychiatry Rep.

[100] Vicente B, Kohn R, Rioseco P, Saldivia S, Baker C, Torres S. Population prevalence of psychiatric disorders in Chile: 6-month and 1-month rates. Braz J Psychiatry. 2004;184:299–305. 10.1192/bjp.184.4.299 15056573

[101] Ministerio del Interior y Seguridad Pública: Migración en Chile 2005 - 2014. In: Migraciones Chile. Edited by y SEdDdE. Retrieved from: https://www.extranjeria.gob.cl/media/2016/06/Anuario.pdf. Accessed 23 May 2023.

