# Supplementary material for: Common mental disorders in Peruvian immigrant in Chile: a comparison with the host population
Source: BMC Public Health. 2023 Jun 30;23:1274. doi: 10.1186/s12889-023-15793-7 (PMC10314508; doi:10.1186/s12889-023-15793-7)
Supplement: Supplementary file 1 — Additional file 1. [file 12889_2023_15793_MOESM1_ESM.pdf]

Supplementary Online Content

eFigure 1. Three-stage random sampling procedure to select adults born in Peru in Santiago and Recoleta

eFigure 2. Final sample disposition and response rate

eTable 1. Findings from US population-based studies of prevalence of depressive disorders among immigrants

eTable 2. Findings from US population-based studies of prevalence of any anxiety disorder among immigrants

eTable 3. Weights for each category of participants (sex, age group and country of birth)

eTable 4. Item total correlations and Cronbach's alpha coefficients

eTable 5. Mismatch between pre-migratory expectations and post-migratory achievements by sex

eTable 6. Sex differences in one-week prevalence of CIS-R Common Mental Disorders by immigrant group (Odds Ratios [ORs] and 95% Confidence Intervals [CIs])

eTable 7. Mental health service utilization among cases of *any CMD* by sex and immigrant status (Odds Ratios [ORs] and 95% Confidence Intervals [CIs])

eTable 8. Differences in CIS-R total scores among cases of any CMD by mental health service utilization

eTable 9. Findings from Peruvian population-based surveys of point and 1-month prevalence of depressive and anxiety disorders in the general population

eTable 10. Findings from Latin America population-based studies of prevalence of one-month ICD-10 depressive and anxiety disorders in the general population using structured interviews

eTable 11. Findings from international population-based studies of prevalence of past-week ICD-10 common mental disorders (CIS-R)

eFigure 1. Three-stage random sampling procedure to select adults born in Peru in Santiago and Recoleta

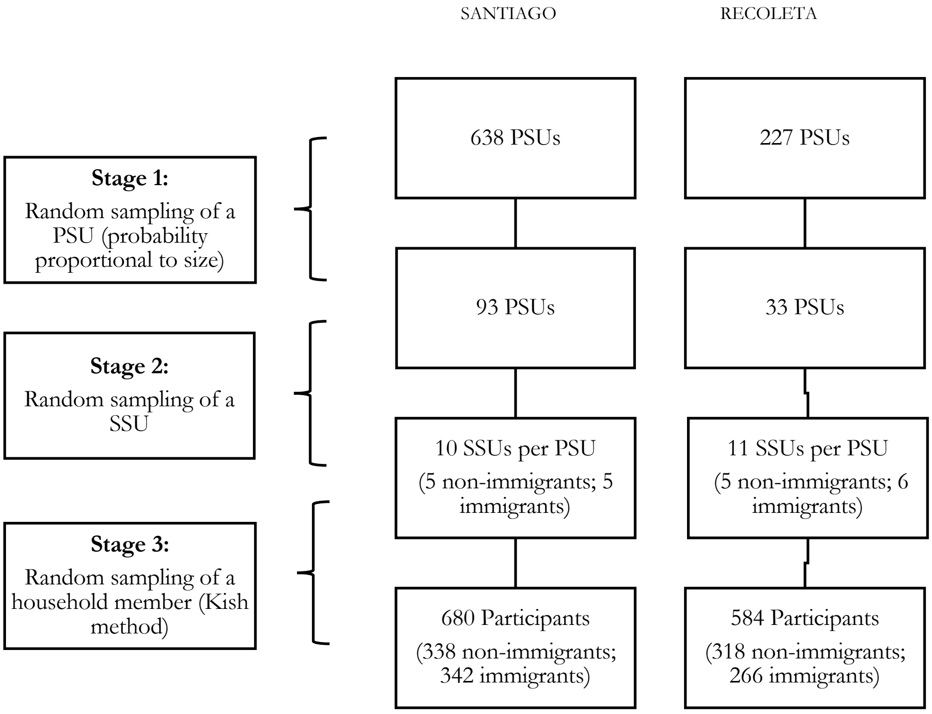

PSU: primary sampling unit (conglomerate of 200 households on average)
SSU: secondary sampling unit (individual household within each conglomerate)
Final sampling unit (household members meeting the study's inclusion criteria)

eFigure 2. Final sample disposition and response rate

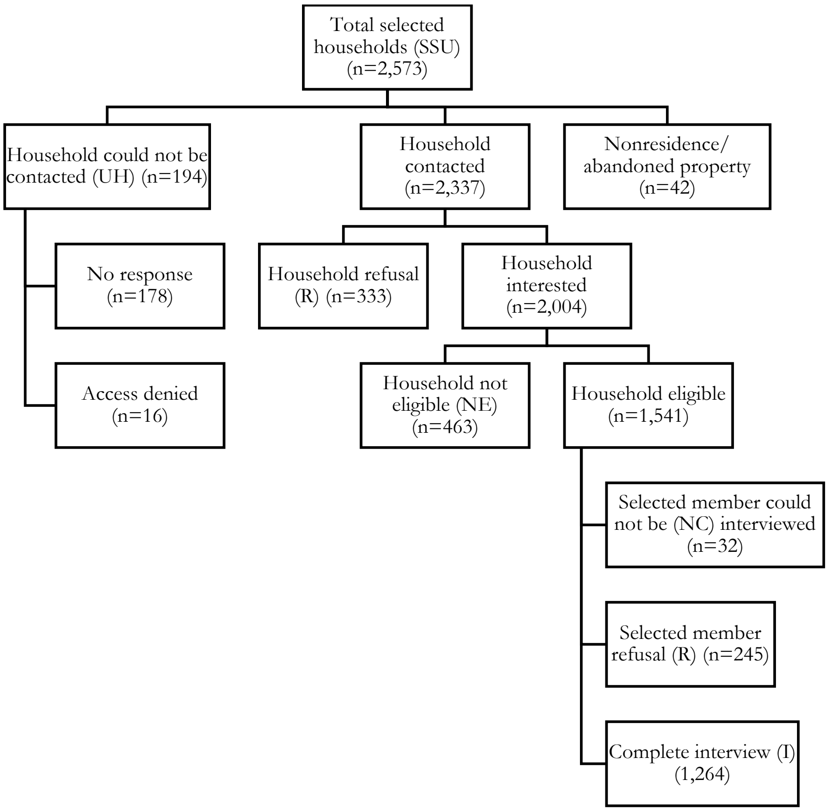

when using the following formula:

$$Response\ Rate = \frac{I}{I + R + NC + UH}$$

where:
I = Complete Interviews; *R* = Refusal; *NC* = Non-Contact; *UH* = Unknown Households

eTable 1. Findings from US population-based studies of prevalence of depressive disorders[a] among migrants

Author	Study	Assess. Method	Diag. criteria	Age	Immigrant group	Sample size	Comparison non-immigrant group(s)	Prev[b]	Sex[c]	Prevalence % [SE] / Odds Ratios (95% CI) Immigrants	Non-immigrants
(Burnam et al., 1987)	LAECA	DIS	DSM-III	18+	Mexican	707	US-born Mexican (n=537)	L	All	3.3%	6.9%*
(Vega et al., 1998)	MAPSS	CIDI	DSM-III-R	18-59	Mexican	1,834	US-born Mexican (n=1,178)	L	All	5.2% [0.8]	14.8% [1.7]
(Grant et al., 2004)	NESARC	CIDI	DSM-IV	18+	Mexican	2,227	US-born Mexican (n=2,331)	L	All	7.7% [0.6]	15.2% [1.4]
					Non-Hispanic white	1,541	US-born non-Hispanic white (n=22,262)	L	All	12.0% [1.0]	18.2% [0.4]
(Breslau & Chang, 2006)	NESARC	CIDI	DSM-IV	18+	Asian	964	US-born Asian (n=282)	L	All	8.2% [1.2]	11.7% [1.8] ^{ns}
(Williams et al., 2007)	NSAL	CIDI	DSM-IV	18+	African American and Caribbean Black	1,160	US-born African and Caribbean Black (n=440)	12-m	M	OR=0.35 (0.09-1.28)^{ns}	1.0 [ref]
									F	OR=1.04 (0.48-2.28)^{ns}	1.0 [ref]
								L	M	OR=0.32 (0.12-0.83)*	1.0 [ref]
									F	OR=0.63 (0.34-1.19)^{ns}	1.0 [ref]
(Alegría et al., 2008)	NCS-R	CIDI	DSM-IV	18+	Non-Latino White	134	US-born non-Latino white (n=4,088)	L	All	17.5% (11.3-23.9)	26.9^ (24.2-29.8)***
(John et al., 2012)	NLAAS	CIDI	DSM-IV	18+	Asian	1,193	US-born Asian (n=335)	12-m	All	5.0%	8.0%**
(Takeuchi et al., 1998)	NLAAS	CIDI	DSM-IV	18+	Asian	1,639	US-born Asian (n=335)	12-m	M	OR=1.68 (0.76-3.70)^{ns}	1.0 [ref]
									F	OR=0.52 (0.25-1.10)^{ns}	1.0 [ref]
								L	M	OR=0.91 (0.48-1.73)^{ns}	1.0 [ref]
									F	OR=0.50 (0.33-0.76)**	1.0 [ref]
(Alegría et al., 2008)	NLAAS	CIDI	DSM-IV	18+	All Latino	1,630	US-born Latino (n=924)	L	All	13.4% (11.6-15.4)	18.6% (16.3-15.4)***
					Puerto Rican	217	US-born Puerto Rican (n=278)	L	All	17.6% (14.-21.6)	20.2% (16.2-24.2)^{ns}
					Cuban	501	US-born Cuban (n=76)	L	All	18.5% (14.5-22.8)	17.9% (11.9-25.1)^{ns}
					Mexican	488	US-born Mexican (n=380)	L	All	11.8% (9.1-14.5)	19.2% (15.7-22.7)***
					Other Latino	423	US-born Other Latino (n=190)	L	All	14.1% (10.9-17.4)	16.2% (12.2-20.1)^{ns}

Studies are ordered by year of survey

Abbreviations: Composite International Diagnostic Interview (CIDI); Diagnostic Interview Schedule (DIS); Los Angeles Epidemiologic Catchment Area (LAECA); Mexican American Prevalence and Services Survey (MAPPS); National Comorbidity Survey Replication (NCS-R); National Epidemiologic Survey on Alcohol and Related Conditions (NESARC); National Latino and Asian American Study (NLAAS); National Survey of American Life (NSAL); United States (US)

[a] Major Depressive Disorder or Any depressive disorder; [b] Prevalence observation period: Lifetime (L), 12-months (12-m); [c] Male (M), Female (F)

*$p \leq 0.05$; ** $p \leq 0.01$; *** $p \leq 0.001$; ns (not significant)

eTable 2. Findings from US population-based studies of prevalence of any anxiety disorder among migrants

Author	Study	Assess. Method	Diag. criteria	Age range	Immigrant group[a]	Sample size	Comparison non-immigrant group(s)	Prev[b]	Sex[c]	Prevalence % [SE] / Odds Ratios (95% CI)	
										Immigrants	Non-immigrants
(Karno et al., 1989)	LAECA	DIS	DSM-III	18+	Mexican	706	US-born Mexican (n=538)	L	All	13.7% [1.8]	22.6% [2.0]*
							US-born non-Hispanic white (n=1,149)			[ref]	21.9% [1.4]*
(Vega et al., 1998)	MAPSS	CIDI	DSM-III-R	18-59	Mexican	1,834	US-born Mexican (n=1,178)	L	All	13.0% [1.2]	23.2% [1.8]
(Grant et al., 2004)	NESARC	CIDI	DSM-IV	18+	Mexican	2,227	US-born Mexican (n=2,331)	L	All	9.1% [0.9]	16.3% [1.5]
					Non-Hispanic white	1,541	US-born non-hispanic white (n=22,262)	L	All	12.4% [1.0]	18.7% [0.5]
(Breslau et al., 2009)	NESARC	CIDI	DSM-IV	18+	Mexican (early age)	440	US-born Mexican (2,320)	L	All	14.6% [2.1]^{ns}	17.1% [1.5] [ref]
					Mexican (late age)	1,787				8.5% [1.1]**	
					Cuban (early age)	76	US-born Cuban (n=97)	L	All	10.2% [6.8]^{ns}	15.8% [5.4] [ref]
					Cuban (late age)	277				9.2% [1.9]^{ns}	
					Puerto Rican (early age)	135	US-born Puerto Rican (n=561)	L	All	22.0% [3.7]^{ns}	18.7% [2.2]
					Puerto Rican (late age)	299				22.6% [3.5]^{ns}	[ref]
					Central/South Am. (early age)	140	US-born Central/South (n=139)	L	All	10.1% [3.0]*	24.3% [5.2]
					Central/South Am. (late age)	755				8.8% [1.0]*	[ref]
					Western European (early age)	227	US-born Western Europeans (n=15,564)	L	All	19.1% [3.1]^{ns}	19.5% [0.5]
					Western European (late age)	387				21.0% {2.4]^{ns}	[ref]
					Eastern European (early age)	42	US-born Western Europeans (n=2,037)	L	All	17.2% [5.8]^{ns}	19.2% [1.2]
					Eastern European (late age)	209				8.1% [2.0]**	[ref]
					Afro/Caribbean (early age)	153	US-born Africa/ Caribbean (n=8,326)	L	All	13.5% [2.6] ^{ns}	15.4% [0.7]
					Afro/Caribbean (late age)	613				7.7% [1.4]*	[ref]
(Breslau & Chang, 2006)	NESARC	CIDI	DSM-IV	18+	Asian	964	US-born Asian (n=282)	L	All	9.1% [1.3]	17.8% [3.5] ^{ns}
(Breslau & Chang, 2006)	NESARC	CIDI	DSM-IV	18+	Asian (early age)	162	US-born Asian (n=282)	L	All	OR=0.75 (0.45-1.35)	1.0 [ref]
					Asian (late age)	792				OR=0.47 (0.27-0.80)	
(Alegría et al., 2008)	NCS-R	CIDI	DSM-IV	18+	Non-Latino White	134	US-born non-Latino white (n=4,088)	L	All	23.3% (17.8-29.1)	30.8% (28.0-33.7)**
(Takeuchi et al., 1998)	NLAAS	CIDI	DSM-IV	18+	Asian	1,639	US-born Asian (n=335)	12-m	M	OR=0.89 (0.48-1.64)^{ns}	1.0 [ref]
									F	OR=0.38 (0.17-0.84)*	1.0 [ref]
								L	M	OR=0.90 (0.46-1.73)^{ns}	1.0 [ref]
									F	OR=0.47 (0.26-0.85)**	1.0 [ref]
(John et al., 2012)	NLAAS	CIDI	DSM-IV	18+	Asian	1,193	US-born Asian (n=335)	12-m	All	6.0% [ref]	9.0%**
(Alegría et al., 2008)	NLAAS	CIDI	DSM-IV	18+	All Latino	1,630	US-born Latino (n=924)	L	All	15.2% (13.2-17.3)	18.9% (16.2-21.5)**
					Puerto Rican	217	US-born Puerto Rican (n=278)	L	All	21.8% (16.9-26.7)	21.6% (17.9-25.7)^{ns}
					Cuban	501	US-born Cuban (n=76)	L	All	14.1% (10.8-17.5)	16.7% (10.7-22.6)^{ns}
					Mexican	488	US-born Mexican (n=380)	L	All	14.2% (11.3-17.1)	20.0% (16.2-23.8)**
					Other Latino	423	US-born Other Latino (n=190)	L	All	16.0% (12.5-19.5)	14.1% (10.3-18.8)^{ns}

Studies are ordered by year of survey

Abbreviations: Composite International Diagnostic Interview (CIDI); Diagnostic Interview Schedule (DIS); Los Angeles Epidemiologic Catchment Area (LAECA); Mexican American Prevalence and Services Survey (MAPPS); National Comorbidity Survey Replication (NCS-R); National Epidemiologic Survey on Alcohol and Related Conditions (NESARC); National Latino and Asian American Study (NLAAS); National Survey of American Life (NSAL); United States (US)

[a] Immigrants who arrived at age 13 or earlier (early age); Immigrants who arrived after age 13 (late age). [b] Prevalence observed period: Lifetime (L), 12-months (12-m); [c] Male (M), Female (F)

*p ≤ 0.05; ** p ≤ 0.01; *** p ≤ 0.001; ns (not significant)

eTable 3. Weights for each category of participants (sex, age group and country of birth)

Sample	Country of birth	Sex	Age group[a]	Observed n[b]	Population N[c]	Expected n[de]	Weighting Factor[f]
Non-immigrant	Chile	Male	15-24	73	1,257,143	84.78	1.16
	Chile	Male	24-34	64	1,188,023	80.12	1.25
	Chile	Male	35-44	50	1,158,885	78.16	1.56
	Chile	Male	45-54	60	811,156	54.71	0.91
	Chile	Male	55-64	50	527,899	35.60	0.71
	Chile	Female	15-24	72	1,224,372	82.57	1.15
	Chile	Female	24-34	76	1,204,892	81.26	1.07
	Chile	Female	35-44	64	1,207,873	81.46	1.27
	Chile	Female	45-54	88	852,513	57.49	0.65
	Chile	Female	55-64	78	575,977	38.84	0.50
Immigrant	Peru	Male	15-24	55	2,496	46.64	0.85
	Peru	Male	24-34	99	5,599	104.62	1.06
	Peru	Male	35-44	86	3,029	56.60	0.66
	Peru	Male	45-54	30	1,190	22.23	0.74
	Peru	Male	55-64	8	396	7.40	0.92
	Peru	Female	15-24	63	4,243	79.28	1.26
	Peru	Female	24-34	125	8,520	159.19	1.27
	Peru	Female	35-44	94	4,825	90.15	0.96
	Peru	Female	45-54	39	2,196	41.03	1.05
	Peru	Female	55-64	19	581	10.86	0.57

a Considering the 2002 Census data is not presented for the 18-24 age range, the 15-24 range was used for the 18-24 age group in our study
b Observed in this study (ISHS)
c Cases in 2002 Chilean population Census[1].
d Calculated using this formula for the 'immigrant' sample:

$$expected_{\_sex\_age_{ij}} = \frac{Number\ of\ ISHS\ participants\ born\ in\ Peru \times frequency\ sex\ i\ in\ age\ group\ j\ born\ in\ Peru\ in\ Census}{Number\ of\ people\ 15-64\ bor[illegible]\ in\ Peru\ in\ Census}$$

where: *i*= men, women; *j*= 1: 15-24 years, 2: 25-34 years, 3: 35-44 years, 4: 45-54 years, 5: 55-64 years

e Calculated using this formula for the 'non-immigrant' sample and adjusted using national level data from the 2002 census:

$$expected_{\_sex\_age_{ij}} = \frac{Number\ ISHS\ participants\ born\ in\ Chile \times frequency\ sex\ i\ in\ age\ group\ j\ in\ Census}{Number\ of\ people\ 15-64\ in\ Census}$$

where: *i*= men, women; *j*= 1: 15-24 years, 2: 25-34 years, 3: 35-44 years, 4: 45-54 years, 5: 55-64 years

f Calculated for each category using this formula and adjusted using national level data from respondents to the 2002 census reporting Peru as their country of birth[2]:

$$w_{\_sex\_age}{}_{ij}^{k} = \frac{expected\ number\ of\ cases\ sex\ i\ in\ age\ group\ j\ in\ k}{observed\ number\ of\ cases\ sex\ i\ in\ age\ group\ j\ in\ k}$$

where: *k*= Chile, Peru; *i*= men, women; *j*= 1: 15-24 years, 2: 25-34 years, 3: 35-44 years, 4: 45-54 years, 5: 55-64 years

1 http://espino.ine.cl/cgibin/RpWebEngine.exe/PortalAction?&BASE=CPCHL2KREG
2 http://www.eclac.cl/migracion/imila/seleccion.asp?parametro=CL02|R|CHILE%202002

eTable 4. Item total correlations and Cronbach's alpha coefficients

Questionnaire/Interview<br>Subscale	Present study: Immigrants<br>n	Present study: Immigrants<br>Alpha Cronbach	Present study: Non-immigrants<br>n	Present study: Non-immigrants<br>Alpha Cronbach	Validation: Spanish (Spain)<br>(Bellón et al., 1996)	Validation: Spanish (Spain)<br>(Moreno-Jiménez et al., 1997)	Validation: Spanish (Peru)<br>(De Silva et al., 2007)	Validation: Spanish (Chile)<br>(Lewis et al., 1992)
Duke-UNC Functional Social Support (Duke-UNC)	569	0.90	617	0.93	0.92			
Orientation to Life Questionnaire (OLQ-13)	583	0.78	625	0.79		0.83		
Comprehensibility	592	0.62	640	0.65		0.64		
Manageability	598	0.48	638	0.51		0.69		
Meaningfulness	592	0.52	642	0.44		0.74		
Short Adapted Social Capital Assessment Tool (SASCAT)							Only cognitive validation available	
Cognitive social capital	587	0.61	634	0.71				
Revised Clinical Interview Schedule (CIS-R)	542	0.83	578	0.82			NA	0.88

Not Available (NA)

eTable 5. Mismatch between pre-migratory expectations and post-migratory achievements by sex

	Men		Women		All	
Dimension of expectations achieved	n	%	n	%	n	(%)
Work						
Perfectly/partially achieved	245	(89.7)	265	(80.5)	510	(84.7)
Poorly/not at all achieved	28	(10.3)	64	(19.5)	92	(15.3)
Income						
Perfectly/partially achieved	240	(87.9)	277	(83.9)	517	(85.7)
Poorly/not at all achieved	33	(12.1)	53	(16.1)	86	(14.3)
Family						
Perfectly/partially achieved	229	(83.9)	277	(83.9)	506	(83.9)
Poorly/not at all achieved	44	(16.1)	53	(16.1)	97	(16.1)
Health						
Perfectly/partially achieved	248	(91.2)	282	(85.5)	530	(88.0)
Poorly/not at all achieved	24	(8.8)	48	(14.5)	72	(12.0)
Friends						
Perfectly/partially achieved	243	(88.4)	260	(78.8)	503	(83.1)
Poorly/not at all achieved	32	(11.6)	70	(21.2)	102	(16.9)

eTable 6. Sex-differences in one-week prevalence of ICD-10 Common Mental Disorders (CIS-R) (Odds Ratios [ORs] and 95% Confidence Intervals [CIs])

	Men			Women				
	n	%	(95% CI)	n	%	(95% CI)	OR (95% CI)†	*p* value
Immigrants								
Depressive episode[a]	2	0.8	(0.0-1.9)	29	9.5	(6.2-12.8)	13.4 (2.9-63.5)	0.001
Any anxiety disorder[b]	11	4.3	(1.8-6.9)	37	11.4	(7.9-14.9)	2.8 (1.4-5.8)	0.005
Mixed anxiety and depressive disorder[c]	22	9.0	(5.4-12.7)	64	21.7	(16.9-26.5)	2.8 (1.6-4.8)	<0.001
Any CMD[d]	34	14.0	(9.6-18.5)	117	38.7	(33.1-44.2)	4.0 (2.7-6.3)	<0.001
Non-immigrants								
Depressive episode	9	3.6	(1.2-6.0)	36	10.2	(6.9-13.6)	3.0 (1.5-6.1)	0.002
Any anxiety disorder	16	5.9	(3.0-8.7)	50	14.0	(10.2-17.8)	2.6 (1.5-4.6)	0.001
Mixed anxiety and depressive disorder	41	16.6	(11.8-21.4)	77	23.2	(18.5-28.0)	1.9 (1.0-2.3)	0.052
Any CMD	61	24.2	(18.8-29.7)	150	44.8	(39.2-50.4)	2.6 (1.8-3.8)	<0.001

[a] Includes any depressive episode in ICD-10 (f32.00, f32.01, f32.1, f32.11 or f32.2)
[b] Includes generalized anxiety disorder (f41.1), obsessive–compulsive disorder (f42.0), panic disorders (f41.0), phobia (f40.00, f40.01, f40.1, f40.2)
[c] Corresponds to ICD-10 f41.2 and is defined as a total CIS-R score greater or equal to 12 but not meeting criteria for any specific anxiety disorder (b) or depressive episode (a)
[d] Includes ICD-10 depressive episode (as in a), any ICD-10 anxiety disorder (as in b) and Mixed anxiety and depressive disorder (as in c)
Frequencies unweighted, percentages and 95% CI weighted for age and sex using 2002 Chilean Population Census
† Adjusted for age (ref: men)
Abbreviations: Revised Clinical Interview Schedule (CIS-R); Common Mental Disorder (CMD)

eTable 7. Mental health service utilization among cases of *any CMD* (CIS-R) by sex and immigrant status (Odds Ratios [ORs] and 95% Confidence Intervals [CIs])

	Immigrants			Non-immigrants				
	n	%	(95% CI)	n	%	(95% CI)	OR (95% CI) †	*p* value
Sex								
Women	24	20.3	(12.9-27.7)	39	24.4	(17.3-31.5)	1.2 (0.7-2.3)	0.568
Men	4	11.6	(0.6-22.6)	9	16.3	(6.4-26.2)	1.6 (0.4-6.8)	0.484
All	28	18.6	(12.3-25.0)	48	21.6	(15.8-27.4)	1.3 (0.7-2.3)	0.371

Frequencies unweighted, percentages weighted for age and sex using 2002 Chilean Population Census
† Adjusted for age and sex (ref: immigrants)
Abbreviations: Revised Clinical Interview Schedule (CIS-R); Common Mental Disorder (CMD)

eTable 8. Differences in CIS-R total scores among cases of *any CMD* by mental health service utilization

	Contact			No utilization				
	n	Median	(IQR)	n	Median	(IQR)	U	*p* value
Immigrants	26	18.2	(12.0-25.0)	116	18.0	(14.3-24.0)	1217.5	0.124
Non-immigrants	45	22.0	(16.5-26.5)	156	18.0	(14.0-24.0)	2660.5	0.013
All	71	20.0	(14.0-26.0)	272	18.0	(14.0-24.0)	8823.5	0.262

Frequencies unweighted, medians weighted for age and sex using 2002 Chilean Population Census
Abbreviations: Revised Clinical Interview Schedule (CIS-R); Common Mental Disorder (CMD)

Table 9. Findings from Peruvian population-based surveys of point and 1-month prevalence of depressive and anxiety disorders in the general population

									Prevalence % (95% CI) [SE}				
									Depression				
Author	Country (region)	POP[a]	Fieldwork	Age range	Sample size	Classific. System	Assess. Method	Prevalence	Depressive Disorder[b]	Mild, moderate or severe depressive episode[c]	Moderate or severe depressive episode	MDD	Any anxiety disorder[d]
Present study	Chile (Santiago)	I	2011	18-64	608	ICD-10	CIS-R	1-week		6.2 (4.1-8.3)			8.6 (6.2-11.0)
(Bromet et al., 2018)	Peru (national)	G	2004-2005	18-65	3,930	DSM-IV	CIDI	1-month				0.7 (0.4-1.0)	
(Instituto Especializado de Salud Mental, 2002)	Peru (Metropolitan Lima)	G	2002	18-60	3,895	ICD-10	MINI	Point	6.7 (5.6-7.9)		6.6 (5.6-7.9)		14.6 (12.7-16.7)
(Instituto Especializado de Salud Mental, 2003)	Peru (Sierra)	G	2003	18-60	3,909	ICD-10	MINI	Point	3.7 (3.0-4.4)		3.5 (2.9-4.2)		3.5 (2.8-4.4)
(Instituto Especializado de Salud Mental, 2005)	Peru (Selva Peruana)	G	2004	18-60	5,857	ICD-10	MINI	Point	2.5 (1.9-2.9)		2.1 (1.6-2.9)		3.7 (2.9-4.6)
(Instituto Especializado de Salud Mental, 2006)	Peru (Fronteras)	G	2005	18-60	6,555	ICD-10	MINI	Point	3.1 (2.5-3.8)		2.9 (2.3-3.5)		4.7 (3.9-5.6)
(Instituto Especializado de Salud Mental, 2007)	Peru (Costa Peruana)	G	2006	18-60	2,536	ICD-10	MINI	Point	1.6 (1.1-2.1)		1.5 (1.1-2.1)		1.8 (1.2-2.7)
(Instituto Especializado de Salud Mental, 2008)	Peru (Rural Lima)	G	2007	18-60	3,031	ICD-10	MINI	Point	1.9 (1.2-3.0)		1.5 (0.9-2.6)		2.6 (1.6-4.1)
(Instituto Especializado de Salud Mental, 2009)	Peru (Rural Sierra)	G	2008	18-60	1,746	ICD-10	MINI	Point	1.4 (0.9-2.1)		1.3 (0.8-2.9		1.7 (1.1-2.5)
(Instituto Especializado de Salud Mental, 2011)	Peru (Abancay)	G	2009	18-60	2,331	ICD-10	MINI	Point	1.1 (0.7-1.8)		0.6 (0.4-1.0)		2.5 (1.7-3.5)
(Instituto Especializado de Salud Mental, 2012)	Peru (Rural Selva)	G	2010	18-60	4,445	ICD-10	MINI	Point	0.7 (0.4-1.0)		0.8 (0.5-1.4)		1.5 (1.0-2.4)
(Instituto Especializado de Salud Mental, 2013)	Peru (Metropolitan Lima-R)	G	2012	18-60	1,469	ICD-10	MINI	Point	2.8 (2.3-3.5)		1.9		1.9 (1.5-2.4)
(Instituto Especializado de Salud Mental, 2016a)	Peru (Cerro de Pasco)	G	2013	18-60	1,496	ICD-10	MINI	Point	2.5 (1.7-3.5)		2.0		2.7 (1.9-3.7)
(Instituto Especializado de Salud Mental, 2016b)	Peru (Huánuco)	G	2013	18-60	1,724	ICD-10	MINI	Point	2.5 (1.8-3.5)		1.9		2.1 (1.4-3.2)
(Instituto Especializado de Salud Mental, 2019)	Peru (Abancay-R)	G	2016	18-60	608	ICD-10	MINI	Point	3.0 (2.1-4.3)		2.8 (1.9-4.1)		NA

[a] POP (population): Immigrant (I); General (G)
[b] Includes depressive episode and dysthymia
[c] Includes f32.00, f32.01, f32.1, f32.11 or f32.2
[d] Present study includes panic disorder, general anxiety disorder, obsessive compulsive disorder and phobia; Studies conducted by the Instituto Especializado de Salud Mental include agoraphobia, panic disorder, general anxiety disorder, obsessive compulsive disorder, social phobia and post-traumatic stress disorder
Abbreviations: Composite International Diagnostic Interview (CIDI); Clinical Interview Schedule – Revised (CIS-R); Major Depressive Disorder (MDD); Mini-International Neuropsychiatric Interview (MINI); Not available (NA)

eTable 10. Findings from Latin America studies of prevalence of one-month ICD-10 depressive and anxiety disorders in the general population using structured interviews, by sex

Author	Country (Setting)	Survey	Survey Years	Assess. Method	Age range	Sample size	POP[a]	Depression definition	Any anxiety definition	Prevalence % (95% CI) [SE]					
										Depression			Any anxiety disorder		
										Men	Women	All	Men	Women	All
Present study	Chile (Santiago RM)	ISHS	2011	CIS-R	18-64	608	I	Depressive episode (mild, moderate, or severe)	Includes GAD, OCD, PD and phobia	0.8 (0.0-1.9)	9.5 (6.2-12.8)	6.2 (4.1-8.3)	4.3 (1.8-6.9)	11.4 (7.9-14.9)	8.6 (6.2-11.0)
						656	NI			3.6 (1.2-6.0)	10.2 (6.9-13.6)	7.0 (4.9-9.1)	5.9 (3.0-8.7)	14.0 (10.2-17.8)	10.0 (7.6-12.4)
(Andrade et al., 2002)	Brazil (Sao Paulo)	ECA-SP	1994-1996	CIDI	18+	1,464	G	Depressive Episode	Includes AG, OCD, PD, SoP and SP	3.2 [0.9]	5.4 [0.6]	4.5 [0.6]	3.5 [0.8]	7.8 [0.7]	6.0 [0.5]
(Vorcaro et al., 2001)	Brazil (Bambui)	BHS	1996-1997	CIDI	18+	1,041	G	Depressive episode (mild, moderate, or severe) or recurrent depressive disorder		4.0 (2.2-5.8)	11.4 (8.8-13.9)	8.2 (6.5-9.8)			
(Gómez-Restrepo et al., 2004)	Colombia (national)	HHSRS	2000-2001	CIDI	18+	6,116	G	Depressive episode (mild, moderate, or severe)		5.3 (4.4-6.3)	10.2 (9.2-11.1)	8.5 (7.8-9.2)			
(Medina-Mora et al., 2003)	Mexico (national urban)	NSPE	2001-2002	CIDI	18-65	2,432	G	Major depressive episode	Includes AG without panic, OCD, PD, SoP, SP, PTSD	0.2 [0.1]	0.5 [0.1]	0.3 [0.1]	2.2 [0.4]	4.1 [0.5]	3.2 [0.3]
								Minor depressive episode		0.4 [0.1]	0.4 [0.1]	0.4 [0.1]			

Studies are ordered alphabetically by country and by year of survey

[a] POP (population): Immigrant (I); General (G)

Abbreviations: AG (Agoraphobia); BHS (Bambui Health Survey); CIDI (Composite International Diagnostic Interview); CIS-R (Clinical Interview Schedule – Revised); ECA-SP (Epidemiologic Catchment Area Study in the city of Sao Paulo); GAD (General Anxiety Disorder); HHSRS (Health and Health System Responsiveness Survey); ISHS (Inner Santiago Health Study); NSPE (National Survey on Psychiatric Epidemiology); OCD (obsessive compulsive disorder); PD (Panic Disorder); SoP (Social Phobia); SP (Specific Phobia); PTSD (Posttraumatic stress disorder)

eTable 11. Findings from international population-based studies of prevalence of past-week ICD-10 common mental disorders (CIS-R)

						Prevalence %								
						Depressive episode			Any anxiety disorder			Any common mental disorder		
Author	Country	Survey	Age range	Sample size	Population	Men	Women	All	Men	Women	All	Men	Women	All
Present study	Chile (Santiago)	ISHS	18-64	608	Immigrant	0.8	9.5	6.2	4.3	11.4	8.6	14.0	38.6	29.1
				656	Non-immigrant	3.6	10.2	7.0	5.9	14.0	10.0	24.2	44.8	34.7
(Araya et al., 2001)	Chile (Santiago)	SMDS	16-64	3,870	General	2.7	8.0	5.5				17.3	35.2	26.7
(Lam et al., 2015)	China (Hong Kong)	HKMMS	16-75	5,589	General	2.2	3.5	2.9				9.4	16.9	
(Dhadda & Greene, 2018)	England	EMPIRIC	16-74	2,212	Immigrant minorities									16.9
					Non-immigrant minorities									17.4
(Singleton et al., 2003)	Great Britain	APMS 2000	16-74	8,886	General			2.6						16.4
(McManus et al., 2009)	Great Britain	APMS 2007	16-74	7,403	General			2.3						16.2
(McManus et al., 2016)	Great Britain	APMS 2014	16-74	7,546	General			3.3						17.0
(Skapinakis et al., 2013)	Greece		18-70	4,894	General	2.1	3.7	2.9	4.5	8.5	6.5			
(Jenkins et al., 2012)	Kenya		16-65	876	General			0.7				10.9	10.8	10.8
(Krishnaswamy et al., 2012)	Malaysia	MMHS	16+	3,666	General			0.4				3.6	7.0	5.3

Studies are ordered alphabetically by country and by year of survey

Abbreviations: Adult Psychiatric Morbidity Surveys (APMS); Clinical Interview Schedule – Revised (CIS-R); Inner Santiago Health Study (ISHS); Ethnic Minority Psychiatric Illness Rates in the Community (EMPIRIC); Hong Kong Mental Morbidity Survey (HKMMS); Malaysian Mental Health Survey (MMHS); Santiago Mental Disorders Survey (SMDS)

References

Alegría, M., Canino, G., Shrout, P., Woo, M., Duan, N., Vila, D., et al. (2008). Prevalence of mental illness in immigrant and non-immigrant U.S. Latino groups. *The American journal of psychiatry,* 165, 359-369.

Andrade, L., Walters, E.E., Gentil, V., & Laurenti, R. (2002). Prevalence of ICD-10 mental disorders in a catchment area in the city of São Paulo, Brazil. *Soc Psychiatry Psychiatr Epidemiol,* 37, 316-325.

Araya, R., Rojas, G., Fritsch, R., Acuña, J., & Lewis, G. (2001). Common mental disorders in Santiago, Chile: prevalence and socio-demographic correlates. *Br J Psychiatry,* 178, 228-233.

Bellón, J., Delgado, A., Luna, J., & Lardelli, P. (1996). Validez y fiabilidad del cuestionario de apoyo social funcional Duke-UNC-11. *Atención Primaria,* 18, 153-163.

Breslau, J., Borges, G., Hagar, Y., Tancredi, D., & Gilman, S. (2009). Immigration to the USA and risk for mood and anxiety disorders: variation by origin and age at immigration. *Psychological medicine,* 39, 1117-1127.

Breslau, J., & Chang, D. (2006). Psychiatric disorders among foreign-born and US-born Asian-Americans in a US national survey. *Social psychiatry and psychiatric epidemiology,* 41, 943-950.

Bromet, E., Andrade, L., Bruffaerts, R., & Williams, D. (2018). Major Depressive Disorder. In K.M. Scott, P. de Jonge, D.J. Stein, & R.C. Kessler (Eds.), *Mental Disorders Around the World: Facts and Figures from the WHO World Mental Health Surveys* pp. 41-56): Cambridge University Press.

Burnam, M.A., Hough, R.L., Karno, M., Escobar, J.I., & Telles, C.A. (1987). Acculturation and Lifetime Prevalence of Psychiatric Disorders Among Mexican Americans in Los Angeles. *Journal of Health and Social Behavior,* 28, 89-102.

De Silva, M.J., Huttly, S.R., Harpham, T., & Kenward, M.G. (2007). Social capital and mental health: a comparative analysis of four low income countries. *Soc Sci Med,* 64, 5-20.

Dhadda, A., & Greene, G. (2018). 'The Healthy Migrant Effect' for Mental Health in England: Propensity-score Matched Analysis Using the EMPIRIC Survey. *J Immigr Minor Health,* 20, 799-808.

Gómez-Restrepo, C., Bohórquez, A., Pinto Masis, D., Gil Laverde, J.F., Rondón Sepúlveda, M., & Díaz-Granados, N. (2004). [The prevalence of and factors associated with depression in Colombia]. *Rev Panam Salud Publica,* 16, 378-386.

Grant, B.F., Stinson, F.S., Hasin, D.S., Dawson, D.A., Chou, S.P., & Anderson, K. (2004). Immigration and lifetime prevalence of DSM-IV psychiatric disorders among Mexican Americans and non-Hispanic whites in the United States: results from the National Epidemiologic Survey on Alcohol and Related Conditions. *Arch Gen Psychiatry,* 61, 1226-1233.

Instituto Especializado de Salud Mental (2002). Estudio Epidemiológico Metropolitano de Salud 2002. *Anales de Salud Mental,* XVIII (Números 1 y 2), Peru. Retrieved from http://www.insm.gob.pe/investigacion/archivos/estudios/2002-ASM-EESM-M/files/res/downloads/book.pdf.

Instituto Especializado de Salud Mental (2003). Estudio Epidemiológico de Salud Mental en La Sierra Peruana 2003. *Anales de Salud Mental,* XIX (Números 1 y 2), Peru. Retrieved from https://www.insm.gob.pe/investigacion/archivos/estudios/2003-ASM-EESM-SP/files/res/downloads/book.pdf.

Instituto Especializado de Salud Mental (2005). Estudio Epidemiológico en Salud Mental en la Selva Peruana 2004. *Anales de Salud Mental,* XXI (Números 1 y 2), Peru. Retrieved from https://www.insm.gob.pe/investigacion/archivos/estudios/2004-ASM-EESM-SP/files/res/downloads/book.pdf.

Instituto Especializado de Salud Mental (2006). Estudio Epidemiológico de Salud Mental en Fronteras 2005. *Anales de Salud Mental,* XXII (Números 1 y 2), Peru. Retrieved from https://www.insm.gob.pe/investigacion/archivos/estudios/2005-ASM-EESM-F/files/res/downloads/book.pdf.

Instituto Especializado de Salud Mental (2007). Estudio Epidemiológico de Salud Mental en la Costa Peruana 2006. *Anales de Salud Mental,* XXIII (Números 1 y 2), Peru. Retrieved from https://www.insm.gob.pe/investigacion/archivos/estudios/2006-ASM-EESM-CP/files/res/downloads/book.pdf.

Instituto Especializado de Salud Mental (2008). Estudio Epidemiológico de Salud Mental en Lima Rural 2007. *Anales de Salud Mental,* XXIV (Números 1 y 2), Peru. Retrieved from http://www.insm.gob.pe/investigacion/archivos/estudios/2007-ASM-EESM-LR/files/res/downloads/book.pdf.

Instituto Especializado de Salud Mental (2009). Estudio Epidemiológico de Salud Mental en La Sierra Rural 2008. *Anales de Salud Mental,* XXV (Números 1 y 2), Peru. Retrieved from https://cdn.www.gob.pe/uploads/document/file/389689/Estudio_epidemiol%C3%B3gico_de_salud_mental_en_la_sierra_rural_2008._Informe_general20191016-26158-b7ip7w.pdf.

Instituto Especializado de Salud Mental (2011). Estudio Epidemiológico de Salud Mental en la ciudad de Abancay 2010. *Anales de Salud Mental,* XXVII (Suplemento 1), Peru. Retrieved from

http://www.insm.gob.pe/investigacion/archivos/estudios/2010-asm-eesm-a/files/res/downloads/book.pdf.

Instituto Especializado de Salud Mental (2012). Estudio Epidemiológico de Salud Mental en la Selva Rural 2009. . *Anales de Salud Mental,* XXXVIII (Suplemento 2) Retrieved from http://www.insm.gob.pe/investigacion/archivos/estudios/2009-ASM-EESM-SR.pdf.

Instituto Especializado de Salud Mental (2013). Estudio Epidemiológico de Salud Mental en Lima Metropolitana y Callao-Replicación 2012. *Anales de Salud Mental,* XXIX (Suplemento 1), Peru. Retrieved from http://www.insm.gob.pe/investigacion/archivos/estudios/2012%20ASM%20-EESM%20-LM.pdf.

Instituto Especializado de Salud Mental (2016a). Estudio Epidemiológico de Salud Mental en la Ciudad de Cerro de Pasco 2013. *Anales de Salud Mental,* XXXII (Número 1), Peru. Retrieved from https://www.insm.gob.pe/investigacion/archivos/estudios/2021/Vol%20XXXII%202016%20nro1%20EESM_ciudad%20de%20Cerro%20de%20Pasco%202013%20(1).pdf.

Instituto Especializado de Salud Mental (2016b). Estudio Epidemiológico de Salud Mental en la Ciudad de Huánuco 2013. *Anales de Salud Mental,* XXXII (Número 2), Peru. Retrieved from https://www.insm.gob.pe/investigacion/archivos/estudios/2021/Vol%20XXXII%202016%20nro2%20EESM_ciudad%20de%20Huanuco%202013%20(1).pdf.

Instituto Especializado de Salud Mental (2019). Estudio Epidemiológico de Salud Mental Comparativo Ciudad de Abancay 2016-2016. Informe General del Adulto. *Anales de Salud Mental,* XXXV (Número 2), Peru. Retrieved from https://www.insm.gob.pe/investigacion/archivos/estudios/_notes/Vol%20XXXV%202019%20Nro2%20EESM_comparativo%20ciudad%20de%20Abancay%20%202010-2016.pdf.

Jenkins, R., Njenga, F., Okonji, M., Kigamwa, P., Baraza, M., Ayuyo, J., et al. (2012). Prevalence of common mental disorders in a rural district of Kenya, and socio-demographic risk factors. *International journal of environmental research and public health,* 9, 1810-1819.

John, D.A., de Castro, A.B., Martin, D.P., Duran, B., & Takeuchi, D.T. (2012). Does an immigrant health paradox exist among Asian Americans? Associations of nativity and occupational class with self-rated health and mental disorders. *Social Science & Medicine,* 75, 2085-2098.

Karno, M., Golding, J.M., Burnam, M.A., Hough, R.L., Escobar, J.I., Wells, K.M., et al. (1989). Anxiety disorders among Mexican Americans and non-Hispanic whites in Los Angeles. *J Nerv Ment Dis,* 177, 202-209.

Krishnaswamy, S., Subramaniam, K., Jemain, A.A., Low, W.Y., Ramachandran, P., Indran, T., et al. (2012). Common mental disorders in Malaysia: Malaysian mental health survey, 2003–2005. *Asia-Pacific Psychiatry,* 4, 201-209.

Lam, L.C., Wong, C.S., Wang, M.J., Chan, W.C., Chen, E.Y., Ng, R.M., et al. (2015). Prevalence, psychosocial correlates and service utilization of depressive and anxiety disorders in Hong Kong: the Hong Kong Mental Morbidity Survey (HKMMS). *Soc Psychiatry Psychiatr Epidemiol,* 50, 1379-1388.

Lewis, G., Pelosi, A.J., Araya, R., & Dunn, G. (1992). Measuring psychiatric disorder in the community: a standardized assessment for use by lay interviewers. *Psychological medicine,* 22, 465-486.

McManus, S., Bebbington, P., Jenkins, R., & Brugha, T. (2016). *(eds.) Mental Health and Wellbeing in England: Adult Psychiatric Morbidity Survey 2014*: The Health & Social Care Information Centre, Social Care Statistics. Retrieved from: https://www.gov.uk/government/statistics/adult-psychiatric-morbidity-survey-mental-health-and-wellbeing-england-2014.

McManus, S., Meltzer, H., Brugha, T., Bebbington, P., & Jenkins, R. (2009). *(eds.) Adult psychiatric morbidity in England, 2007: Results of a household survey.*: The Health & Social Care Information Centre, Social Care Statistics. Retrieved from: https://files.digital.nhs.uk/publicationimport/pub02xxx/pub02931/adul-psyc-morb-res-hou-sur-eng-2007-rep.pdf.

Medina-Mora, M.E., Borges, G., Lara, C., Benjet, C., Jaimes, J., Fleiz, C., et al. (2003). Prevalence of mental disorders and use of services: Results from the Mexican National Survey of Psychiatric Epidemiology. *Salud Mental,* 26, 1-16.

Moreno-Jiménez, B., Alonso, M., & Álvarez, E. (1997). Sentido de Coherencia, Personalidad Resistente, autoestima y salud. *Revista de Psicología de la Salud,* 9, 115-138.

Singleton, N., Bumpstead, R., O'Brien, M., Lee, A., & Meltzer, H. (2003). *Psychiatric morbidity among adults living in private households, 2000.* Retrieved from: https://www.researchgate.net/publication/266299241_Adult_psychiatric_morbidity_in_England_2007_Results_of_a_household_survey.

Skapinakis, P., Bellos, S., Koupidis, S., Grammatikopoulos, I., Theodorakis, P.N., & Mavreas, V. (2013). Prevalence and sociodemographic associations of common mental disorders in a nationally representative sample of the general population of Greece. *BMC Psychiatry,* 13, 163.

Takeuchi, D., Chung, R., Lin, K., Shen, H., Kurasaki, K., Chun, C., et al. (1998). Lifetime and Twelve Month Prevalence Rates of Major Depressive Episodes and Dysthmia Among Chinese Americans in Los Angeles. *The American journal of psychiatry,* 155, 1407-1414.

Vega, W., Kolody, B., Aguilar-Gaxiola, S., Alderete, E., Catalano, R., & Caraveo-Anduaga, J. (1998). Lifetime Prevalence of DSM-III-R Psychiatric Disorders Among Urban and Rural Mexican Americans in California. *Archives of General Psychiatry,* 55, 771-778.

Vorcaro, C.M., Lima-Costa, M.F., Barreto, S.M., & Uchoa, E. (2001). Unexpected high prevalence of 1-month depression in a small Brazilian community: the Bambuí Study. *Acta Psychiatr Scand,* 104, 257-263.

Williams, D.R., Haile, R., González, H.M., Neighbors, H., Baser, R., & Jackson, J.S. (2007). The mental health of Black Caribbean immigrants: results from the National Survey of American Life. *American journal of public health,* 97, 52-59.
